# Long noncoding RNA HEGBC promotes tumorigenesis and metastasis of gallbladder cancer via forming a positive feedback loop with IL-11/STAT3 signaling pathway

**DOI:** 10.1186/s13046-018-0847-7

**Published:** 2018-08-07

**Authors:** Liang Yang, Qingxiang Gao, Xiaoxiong Wu, Feiling Feng, Kaiyun Xu

**Affiliations:** 1 Department of Radiotherapy, Eastern Hepatobiliary Surgery Hospital, Shanghai, China; 2 Department of Biliary Branch, Eastern Hepatobiliary Surgery Hospital, Shanghai, China; 3 Department of Interventional Therapy with Tumor, Seventh People’s Hospital, Shanghai University of TCM, Shanghai, China; 4 Department of emergency, Eastern Hepatobiliary Surgery Hospital, Shanghai, China

**Keywords:** Long noncoding RNA, Gallbladder cancer, Tumorigenesis, Metastasis, IL-11/STAT3 signaling pathway

## Abstract

**Background:**

Gallbladder cancer (GBC) is a highly malignant cancer with poor prognosis. Several long noncoding RNAs (lncRNAs) have been reported to be involved in the tumorigenesis and progression of GBC. However, the expressions, clinical significances, and roles of most other lncRNAs in GBC are still unknown.

**Methods:**

The differentially expressed lncRNAs in GBC were screened through re-analyzing the public available microarray datasets. The expression of lncRNA high expressed in gallbladder cancer (lncRNA-HEGBC) in GBC was measured by qRT-PCR. The correlations between HEGBC with clinicopathological characteristics and prognosis were analyzed by Pearson chi-square test and log-rank test. A series of in vitro and in vivo, gain-of and loss-of function assays were performed to investigate the roles of HEGBC in GBC cell proliferation, apoptosis, migration, tumor growth and metastasis. The interactions between HEGBC and IL-11/STAT3 signaling were explored using chromatin isolation by RNA purification (ChIRP), chromatin immunoprecipitation (ChIP), enzyme linked immunosorbent assay (ELISA), qRT-PCR, western blot, and luciferase reporter assays.

**Results:**

We identified a novel lncRNA HEGBC, which is upregulated in GBC and positively associated with advanced TNM stages and poor prognosis of GBC patients. Overexpression of HEGBC increased GBC cell viability, inhibited GBC cell apoptosis, promoted GBC cell migration, and promoted GBC tumor growth and metastasis in vivo. Conversely, depletion of HEGBC decreased GBC cell viability, promoted GBC cell apoptosis, inhibited GBC cell migration, and inhibited GBC tumor growth and metastasis in vivo. Mechanistic investigations showed that HEGBC bound to the promoter of *IL-11*, increased *IL-11* transcription, induced IL-11 autocrine, and activated IL-11/STAT3 signaling pathway. Furthermore, STAT3 also bound to the promoter of *HEGBC* and activated HEGBC expression. Thus, HEGBC/IL-11/STAT3 formed a positive regulatory loop in GBC. Depletion of IL-11 attenuated the oncogenic roles of HEGBC in GBC.

**Conclusions:**

Our findings identified a novel lncRNA HEGBC, which is upregulated and indicts poor prognosis of GBC. HEGBC exerts oncogenic roles in GBC via forming a positive regulatory loop with IL-11/STAT3 signaling. Our data suggested that HEGBC could be a potential prognostic biomarker and therapeutic target for GBC.

**Electronic supplementary material:**

The online version of this article (10.1186/s13046-018-0847-7) contains supplementary material, which is available to authorized users.

## Background

Gallbladder cancer (GBC) is the most common biliary tract malignant cancer, which is highly lethal and has an extremely poor prognosis [1, 2]. Due to its non-specific symptoms and highly invasive property, most GBC patients are diagnosed at advanced stages [3]. Hence, most GBC patients at advanced stages are not candidates for surgical resection [4]. Unfortunately, until now the only curative treatment for GBC is still surgery [5]. Therefore, the mean survival time for GBC ranges from 13.2 months to 19 months [6]. The poor outcome of GBC and the lack of efficient therapies request better understanding of molecular mechanisms underlying GBC tumorigenesis and metastasis, and developing more efficient targeted therapies for GBC.

Although many aberrantly expressed and mutated molecular events have been identified in GBC, most of these focused on protein-coding genes [7]. Recently, with the great progressions of genome and transcriptome sequencing, many non-protein-coding genes have been identified, which accounts for about 70% of the genome, with only 2% of the genome encoding proteins [8]. Most of these non-protein-coding genes transcribe long non-coding RNAs (lncRNAs) [9]. lncRNA is a class of RNA with limited protein coding potential and more than 200 nucleotides in length [10]. Accumulating evidences revealed that lncRNAs are frequently involved in many pathophysiological processes, including cancers [11–15]. Many lncRNAs are dysregulated in cancers [16–19]. Furthermore, many lncRNAs are revealed to control cell proliferation, cell cycle, cell apoptosis, senescence, cell migration, cell invasion, drug resistance of cancer cells and so on [20–23]. For example, lncRNA-PAGBC is reported to be up-regulated in GBC and promote GBC tumorigenesis via competitively binding miR-133b and miR-511 [24]. LncRNA-CCAT1 is reported to be up-regulated in GBC and promote GBC development via negative regulating miR-218-5p [25]. LncRNA HOXA-AS2 is also up-regulated in GBC and promotes GBC proliferation and epithelial-mesenchymal transition [26]. LncRNA GCASPC is reported to be down-regulated in GBC and inhibit pyruvate carboxylase-dependent cell proliferation of GBC cells [27]. LncRNA H19 is reported to be up-regulated and have oncogenic roles in GBC via modulating miR-342-3p and FOXM1 [28]. In our previous study, we investigated the expression and roles of lncRNA SPRY4-IT1 in GBC and found that lncRNA SPRY4-IT1 is upregulated in GBC and promotes GBC cell proliferation, migration, and invasion [29]. Although several lncRNAs have been reported to be involved in GBC, the expression and roles of most of lncRNAs in GBC are still unclear.

In this study, we searched differentially expressed lncRNAs in GBC via analyzing public available microarray data and identified a novelly differently expressed lncRNA in GBC. We further analyzed the expression, clinical significances, roles, and mechanisms of action of this lncRNA in GBC.

## Methods

### Clinical specimens

A total of 102 pairs of GBC tissues and adjacent non-tumor tissues were obtained from GBC patients with written informed consent who underwent surgery at Eastern Hepatobiliary Surgery Hospital (Shanghai, China). All these GBC patients did not receive any pre-operative treatments. The tissue specimens were confirmed by histopathological diagnosis. All resected specimens were immediately snap-frozen in liquid nitrogen and stored at − 80 °C until RNA extraction. The Review Board of Eastern Hepatobiliary Surgery Hospital reviewed and approved this study.

### Cell lines and treatments

The human non-tumorigenic biliary epithelial cell line H69 and GBC cell lines SGC-996, NOZ, GBC-SD, and EH-GB2 were obtained from the Institute of Biochemistry and Cell Biology of the Chinese Academy of Sciences (Shanghai, China) or maintained in our hospital [30]. The cells were maintained in Dulbecco’s Modified Eagle’s Medium (Gibco BRL, Grand Island, NY, USA) supplemented with 10% fetal bovine serum (Gibco) in a humidified incubator containing 5% CO_2_ at 37 °C. Where indicated, the GBC cells were treated with 5 ng/mL doxorubicin (Selleck, Houston, TX, USA) for 24 h, 5 μM p-STAT3 inhibitor SC144 (Selleck) for 72 h, or 20 ng/mL IL-11 (Gibco) for 72 h.

### Quantitative real-time polymerase chain reaction (qRT-PCR)

Total RNA was extracted from tissues and cells using TRIzol Regent (Invitrogen, Carlsbad, CA, USA) in accordance with the manufacturer’s instruction, followed by being treated with DNase I (Takara, Dalian, China) to remove genomic DNA. Reverse transcription was carried out using the extracted RNA and the M-MLV Reverse Transcriptase (Invitrogen) in accordance with the manufacturer’s instruction. Quantitative real-time polymerase chain reaction (qRT-PCR) analyses were performed using SYBR® Premix Ex Taq™ II (Takara) on ABI StepOnePlus Real-Time PCR System (Applied Biosystems, Foster City, CA, USA) in accordance with the manufacturers’ instructions. The quantification of the expression of RNA was normalized to the expression of β-actin. The expression of RNA was calculated using the comparative Ct method. The sequences of the primers were as follows: for HEGBC, 5’-CACAGGAATCTGAAAAAC-3' (forward) and 5’-TAGTGAGAATCAAAGGCA-3' (reverse); for IL-11, 5’-GCTGCAAGGTCAAGATGGTT-3' (forward) and 5’-GCTGGGTGGCGTTCTATC-3' (reverse); for BCL2, 5’-CTTCGCCGAGATGTCCAG-3' (forward) and 5’-CCCAGCCTCCGTTATCCT-3' (reverse); for Cyclin D1, 5’-TCCTCTCCAAAATGCCAGAG-3' (forward) and 5′- GGCGGATTGGAAATGAACTT -3′ (reverse); for Survivin, 5’-GCAGCCCTTTCTCAAGGACC-3′ (forward) and 5’-AGTGGATGAAGCCAGCCTCG-3′ (reverse); for TSLNC8, 5’-CACCTCCATTCAACCAATAAGC-3′ (forward) and 5’-ACCCTGTCCCCAATAACCC-3′ (reverse); and for β-actin, 5’-GGGAAATCGTGCGTGACATTAAG-3′ (forward) and 5’-TGTGTTGGCGTACAGGTCTTTG-3′ (reverse).

### 5' and 3' rapid amplification of cDNA ends (RACE)

The transcriptional initiation and termination sites of HEGBC were determined using the 5' and 3' rapid amplification of cDNA ends (RACE) assays with the 5′/3’ RACE Kit (Roche, Mannheim, Germany) in accordance with the manufacturer’s instruction. The sequences of the primers for RACE assays were as follows: SP1, 5’-CATCAGCACATAACTCGTCC-3'; SP2, 5’-AGATTCCTGTGCTTGCTTACTC-3′; SP3, 5’-GGCTTCTACACTGCCACCTGC-3'; and SP5, 5’-GCAAGCACAGGAATCTGAAAAAC-3'.

### Plasmids and stable cell lines construction

For construction of HEGBC overexpression plasmid, HEGBC full-length sequences were PCR amplified using Thermo Scientific Phusion Flash High-Fidelity PCR Master Mix (Thermo-Fisher Scientific, Waltham, MA, USA) and subcloned into the *Bam*H I and *Eco*R I sites of the pcDNA3.1 plasmid (Invitrogen), termed as pcDNA3.1-HEGBC. The sequences of the primers were as follows: 5’-CGGGATCCGGGAAATGAGGACCACC-3' (forward) and 5’-GGAATTCAATATGCAAAACTTTACATTTTAGTG-3' (reverse). The empty plasmid pcDNA3.1 was used as negative control. Two pairs of cDNA oligonucleotides repressing HEGBC expression were designed, synthesized, and inserted into the SuperSilencing shRNA expression plasmid pGPU6/Neo (GenePharma, Shanghai, China), termed as shHEGBC-1 and shHEGBC-2. The target sites are 5’-GGAGCTTCCAGAAGTGGTTTC-3' and 5’-GCTGATGAGAGACATGTTTGT-3'. A scrambled shRNA was used as negative control and termed as shControl.

For construction of HEGBC stably overexpressed GBC cells, pcDNA3.1-HEGBC or pcDNA3.1 was transfected into SGC-996 and NOZ cells, and then the cells were selected with 800 μg/mL neomycin for 4 weeks. For construction of HEGBC stably depleted GBC cells, shHEGBC-1, shHEGBC-2, or shControl was transfected into GBC-SD and EH-GB2 cells, and then the cells were selected with 1 μg/mL puromycin for 4 weeks.

### Cell proliferation, apoptosis, and migration assays

Cell proliferation was detected with Glo cell viability assay and Ethynyl deoxyuridine (EdU) incorporation assay. For Glo cell viability assay, 2000 indicated GBC cells were plated into 96-well plates and cultured for indicated time. At each indicated time, the luminescence values were detected using the CellTiter-Glo® Luminescent Cell Viability Assay (Promega, Madison, WI, USA) in accordance with the manufacturer’s instruction. EdU incorporation assay was performed with the EdU kit (Roche) in accordance with the manufacturer’s instruction. Results were acquired using the Zeiss fluorescence photomicroscope (Carl Zeiss, Oberkochen, Germany) and quantified via counting at least five random fields. Cell apoptosis was detected with terminal deoxynucleotidyl transferase (TdT)-mediated dUTP nick end labelling (TUNEL) assay. After being treated with 5 ng/mL doxorubicin for 24 h, indicated GBC cells were used to perform TUNEL assay with the In-Situ Cell Death Detection Kit (Roche) in accordance with the manufacturer’s instruction. Results were acquired using the Zeiss fluorescence photomicroscope (Carl Zeiss) and quantified via counting at least five random fields. Cell migration was detected with transwell assay. For transwell assay, 40,000 indicated GBC cells re-suspended in serum-free media with 1 μg/ml Mitomycin C to inhibit cell proliferation were placed into the upper chamber of transwell insert (8-μm pore size; Millipore, Bedford, MA, USA). Medium containing 10% FBS was added to the lower chamber. After incubation for 48 h, the GBC cells remaining on the upper membrane were removed with cotton wool, and whereas the cells migrating through the membrane were fixed with methanol, stained with 0.1% crystal violet, imaged using the Zeiss fluorescence photomicroscope (Carl Zeiss), and quantified via counting at least five random fields.

### In vivo tumorigenesis and metastasis assays

To establish in vivo tumorigenesis model, 2 × 10^6^ indicted GBC cells in 50 μL phosphate buffered saline mixed with 50% matrigel (Invitrogen) were subcutaneously injected into the flanks of 6 weeks old male athymic nude mice (SLRC Laboratory Animal Center, Shanghai, China). Subcutaneous tumor volumes were detected every 7 days with caliper for 28 days and calculated as *a* × *b*^2^ × 0.5 (*a*, longest diameter; *b*, shortest diameter). At the 28th day after injection, the mice were sacrificed and subcutaneous tumors were resected and weighted. The resected subcutaneous tumors were fixed in formalin, paraffin embedded, deparaffinized, rehydrated, and antigen retrieved. For immunohistochemical staining of Ki67, the sections were incubated with primary antibody for Ki67 (Cell Signaling Technology, Boston, MA, USA), followed by being incubated with a horseradish peroxidase-conjugated secondary antibody (Cell Signaling Technology). Finally, the slides were visualized with 3, 3-diaminobenzidine. For detection of cell apoptosis of subcutaneous tumors, the sections were used to perform TUNEL assay with the In-Situ Cell Death Detection Kit (Roche) in accordance with the manufacturer’s instruction. Liver metastasis model was established with intra-splenic injection of 2× 10^6^ indicated GBC cells. Mice were allowed to grow for 6 weeks, and then the mice were sacrificed, and the livers were resected. The liver metastatic foci number was counted via HE staining. The Review Board of Eastern Hepatobiliary Surgery Hospital reviewed and approved the use of animals.

### Isolation of cytoplasmic and nuclear RNA

Cytoplasmic and nuclear RNA were isolated and purified using the Cytoplasmic & Nuclear RNA Purification Kit (Norgen, Belmont, CA, USA) in accordance with the manufacturer’s instruction. The isolated RNA was detected by qRT-PCR as described above.

### Chromatin isolation by RNA purification (ChIRP) assay

ChIRP assay was performed with the Magna ChIRP RNA Interactome Kit (Millipore) in accordance with the manufacturer’s instruction. Antisense DNA probes against HEGBC were designed and synthesized by Biosearch Probe Designer. The sequences of the probes were as follows: 1, 5'-gaaaccacttctggaagctc-3'; 2, 5'-gcttgcttactcatgtacat-3'; 3, 5'-tgtggtttttcagattcctg-3'; 4, 5'-aagcaggcaaattagtgggc-3'; 5, 5'-aactcgtccttattttagtc-3'; 6, 5'-aacatgtctctcatcagcac-3'; 7, 5'-ttcttttgaactgtgtcaca-3'. ChIRP-derived DNA was quantified using qRT-PCR to detect enrichment of chromatin. The sequences of the primers were as follows: for the promoter of *IL11*, 5’-CTTTGCTTCTCTGGTGTGTC-3' (forward) and 5’-CTGGTGAGGTCATTGGCGT-3' (reverse); for the promoter of *ACTB*, 5’-GGCTGGCTTTGAGTTCCTA-3' (forward) and 5’-CCCACCGTCCGTTGTATGT-3' (reverse).

### **Chromatin** immunoprecipitation (ChIP) **assay**

ChIP assay was performed with the EZ-Magna ChIP A/G (17–10,086, Millipore) and a p-STAT3 antibody (5 μg per reaction; 9131, Cell Signaling Technology, Boston, MA, USA) in accordance with the manufacturer’s instruction. ChIP-derived DNA was quantified using qRT-PCR to detect enrichment of chromatin. The sequences of the primers were as follows: for the − 668 site of *HEGBC* promoter, 5’-CACACTGGATTTGTTTCTG-3' (forward) and 5′-GGGTGGTTGGGTTTTTTTT-3' (reverse); for the − 930 site of *HEGBC* promoter, 5’-CTGCCAACCTGGAAGAAA-3' (forward) and 5’-TTAGGGATTAGGAACCCC-3' (reverse); for the − 1211 site of *HEGBC* promoter, 5’-ATGTAGTATCATGAGCCTGGG-3′ (forward) and 5’-GCAAAGTTATGGAAGCCGTG-3′ (reverse); for the − 1556 site of *HEGBC* promoter, 5’-GCAAAGAGAGGCAGGAGT-3′ (forward) and 5’-TGCTGGGTAAATGAGGACA-3′ (reverse); for the distal non-binding site (negative control, NC) of *HEGBC* promoter, 5’-GTTGTCTCATTGTGTCCC-3′ (forward) and 5’-TGTGTGTTTTTCCCTCTTG-3′ (reverse).

### RNA immunoprecipitation (RIP) assay

RIP assay was performed with the Magna RIP RNA-Binding Protein Immunoprecipitation Kit (Millipore) and p-STAT3 antibody (5 μg per reaction; Cell Signaling Technology), STAT3 antibody (5 μg per reaction; Cell Signaling Technology), RPLP0 antibody (5 μg per reaction; Abcam, Hong Kong, China), or negative control IgG in accordance with the manufacturer’s instruction. RIP-derived RNA was quantified using qRT-PCR to detect enrichment of lncRNAs.

### Enzyme linked immunosorbent assay (ELISA)

IL-11 concentration in the culture medium collected for 48 h from indicated GBC cells were measured with the Human IL-11 ELISA Kit (Dakewei Biotech Company, Shanghai, China) in accordance with the manufacturer’s instruction.

### Western blot analysis

Total proteins were extracted from indicated GBC cells using RIPA buffer (Beyotime, Shanghai, China) and separated by 10% sodium dodecyl sulfate-polyacrylamide gel electrophoresis (SDS-PAGE), followed by being transferred to NC membrane. After being blocked with 5% bovine serum albumin, the membranes were incubated with primary antibodies against p-STAT3 (Cell Signaling Technology), STAT3 (Cell Signaling Technology), or β-actin (Sigma-Aldrich, Saint Louis, MO, USA). After being washed, the membranes were incubated with IRDye 800CW goat anti-rabbit IgG or IRDye 700CW goat anti-mouse IgG (Li-Cor, Lincoln, NE, USA), and detected using Odyssey infrared scanner (Li-Cor).

### Luciferase reporter assays

The promoter of *HEGBC* containing the predicted p-STAT3 binding sites was PCR amplified using Thermo Scientific Phusion Flash High-Fidelity PCR Master Mix (Thermo-Fisher Scientific) and subcloned into the *Kpn* I and *Xho* I sites of the pGL3-basic vector (Promega), termed as pGL3-HEGBC-pro. The sequences of the primers were as follows: 5’-GGGGTACCCTATTGCTGCACTCACACACCC-3′ (forward) and 5’-CCGCTCGAGCGCCAGAGCCCAAGCTATC-3′ (reverse). The empty vector pGL3-basic was used as negative control. The p-STAT3 binding sites mutated *HEGBC* promoter was synthesized by GenScript (Nanjing, China) and subcloned into the *Kpn* I and *Xho* I sites of the pGL3-basic vector, termed as pGL3-HEGBC-pro-mut. The constructed luciferase reporter plasmids were cotransfected with the pRL-TK plasmid expressing renilla luciferase into NOZ cells. 12 h later after transfection, the NOZ cells were treated with 5 μM SC144 or 20 ng/mL IL-11 for 72 h. Then the luciferase activity was measured using Dual-Luciferase® Reporter Assay System (Promega) in accordance with the manufacturer’s instruction.

### Statistical analysis

Statistical analyses were carried out using the GraphPad Prism Software. For comparisons, Wilcoxon signed-rank test, Mann-Whitney test, Pearson chi-square test, Log-rank test, Student’s t test, or Pearson’s correlation analysis was performed as indicated. *P* < 0.05 was regarded as statistically significant.

## Results

### A newly identified lncRNA HEGBC was increased in GBC and associated with poor survival of GBC patients

To search the lncRNAs involved in GBC, we analyzed the expression of lncRNAs in GBC using public available dataset from the National Center for Biotechnology Information (NCBI) Gene Expression Omnibus (GEO) (https://www.ncbi.nlm.nih.gov/geo/). We noted a microarray data comparing the expression of lncRNAs and mRNAs in nine GBC tissues and nine corresponding non-tumor tissues with the GEO accession number GSE76633. We re-analyzed the microarray results and the 60 most differentially expressed lncRNAs and mRNAs are shown in Additional file 1: Table S1. Among the differentially expressed lncRNAs, several lncRNAs have been reported. A new lncRNA ENST00000414772 (LOC401585), which was 5.69-fold higher in GBC tissues than corresponding non-tumor tissues, caught our attention and was named as lncRNA high expressed in gallbladder cancer (lncRNA-HEGBC). The *HEGBC* gene has one transcript in the NCBI database (https://www.ncbi.nlm.nih.gov/; NCBI Reference Sequence: NR_125365.1). The full-length of HEGBC was confirmed using rapid amplification of cDNA ends (RACE) assays (Additional file 2: Figure S1). The Open Reading Frame Finder from NCBI (https://www.ncbi.nlm.nih.gov/orffinder/) failed to predict a protein of more than 19 amino acids. The Coding Potential Calculator (http://cpc.cbi.pku.edu.cn/programs/run_cpc.jsp) also predicted HEGBC as a noncoding RNA with coding potential score − 1.26. Furthermore, RIP assays showed no interaction between HEGBC and ribosomal protein RPLP0, a component of the 60S subunit of ribosome (Additional file 2: Figure S2). These data suggested that HEGBC had no protein-coding capability.

**Fig. 1:**
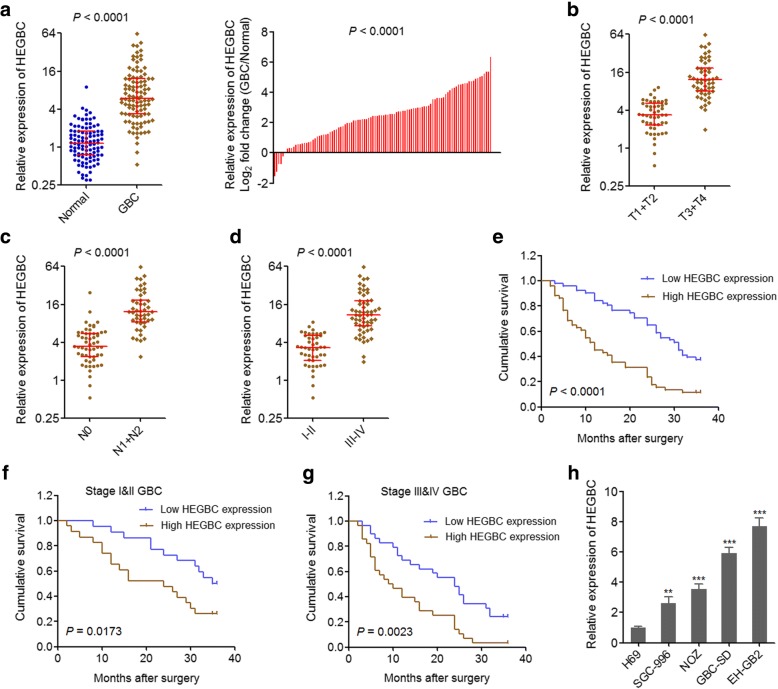
HEGBC was up-regulated in GBC and associated with poor survival of GBC patients. **a** The expression of HEGBC in 102 pairs of GBC tissues and adjacent non-tumor tissues was detected using qRT-PCR. *P* < 0.0001 by Wilcoxon signed-rank test. **b** The expression of HEGBC in 52 GBC extending beyond the gallbladder (T3 + T4) and in 50 GBC only detected in the gallbladder (T1 + T2). *P* < 0.0001 by Mann-Whitney test. **c** The expression of HEGBC in 47 GBC with lymph nodes metastasis (N1 + N2) and in 55 GBC without lymph nodes metastasis (N0). *P* < 0.0001 by Mann-Whitney test. **d** The expression of HEGBC in 57 GBC with advanced clinical stages (III-IV) and in 45 GBC with early clinical stages (I-II). *P* < 0.0001 by Mann-Whitney test. **e** Kaplan-Meier survival analysis of the correlation between HEGBC expression and cumulative survival of the 102 GBC patients. The median expression level of HEGBC was used as the cut-off. *P* < 0.0001 by Log-rank test. **f** Kaplan-Meier survival analysis of the correlation between HEGBC expression and cumulative survival of the 45 stages I-II GBC patients. The median expression level of HEGBC in these 45 GBC was used as the cut-off. *P* = 0.0173 by Log-rank test. **g** Kaplan-Meier survival analysis of the correlation between HEGBC expression and cumulative survival of the 57 stages III-IV GBC patients. The median expression level of HEGBC in these 57 GBC was used as the cut-off. *P* = 0.0023 by Log-rank test. **h** The expression of HEGBC in human non-tumorigenic biliary epithelial cell line H69 and GBC cell lines SGC-996, NOZ, GBC-SD, and EH-GB2 was detected using qRT-PCR. Results are shown as mean ± s.d. of 3 independent experiments. **P < 0.01, ***P < 0.001 by Student’s t test

To confirm the expression pattern of HEGBC in GBC, qRT-PCR was carried out on 102 pairs of GBC tissues and adjacent non-tumor tissues. The results showed that HEGBC was significantly increased in GBC tissues compared with adjacent non-tumor tissues (Fig. 1a). HEGBC was more highly expressed in GBC extending beyond the gallbladder (T3 + T4) than that in GBC only detected in the gallbladder (T1 + T2) (Fig. 1b). HEGBC was also more highly expressed in GBC with lymph nodes metastasis (N1 + N2) than that in GBC without lymph nodes metastasis (N0) (Fig. 1c). Furthermore, HEGBC was more highly expressed in GBC with advanced clinical stages (III-IV) than that in GBC with early clinical stages (I-II) (Fig. 1d). Analyzing of the correlation between the expression of HEGBC and clinicopathologic characteristics of these 102 GBC patients indicated that high HEGBC expression is positively correlated with extending extent (T), lymph node metastasis (N), and TNM stages (Table 1). Kaplan-Meier survival analysis showed that GBC patients with high HEGBC expression had worse survival than those with low HEGBC expression (Fig. 1e). Furthermore, in GBC with clinical stages I-II or III-IV, high HEGBC expression also indicated poor survival (Fig. 1f, g).

**Table 1:** Correlation between HEGBC expression and clinicopathologic characteristics of gallbladder cancer patients

Variables	lncRNA-HEGBC		Chi-square	*P*-value^b^
	Low	High^a^		
All cases	51	51		
Age			0.634	0.426
≥ 60	30	26		
< 60	21	25		
Gender			0.367	0.545
Male	19	22		
Female	32	29		
Serum total bilirubin			0.706	0.401
≥ 17.1 μM	15	19		
< 17.1 μM	36	32		
CA19–9			2.519	0.113
Positive (≥37 U/ml)	23	31		
Negative (< 37 U/ml)	28	20		
Tumor differentiation			0.158	0.691
Well-moderately	29	27		
Poorly	22	24		
T status			62.769	0.000
T1 + T2	45	5		
T3 + T4	6	46		
N status			48.337	0.000
N0	45	10		
N1 + N2	6	41		
TNM stage			54.440	0.000
I-II	41	4		
III-IV	10	47		

^a^The median expression level of HEGBC was used as the cutoff

^b^Pearson chi-square tests were used to analyze the correlation between the expression levels of HEGBC and clinical features

The expression of HEGBC was measured in human non-tumorigenic biliary epithelial cell line H69 and GBC cell lines SGC-996, NOZ, GBC-SD, and EH-GB2 by qRT-PCR. The results showed that HEGBC was also markedly increased in GBC cell lines compared with that in biliary epithelial cell line (Fig. 1h). Collectively, these results showed that HEGBC was upregulated in GBC and associated with poor survival of GBC patients.

### Ectopic expression of HEGBC promoted the proliferation and migration of GBC cells

**Fig. 2:**
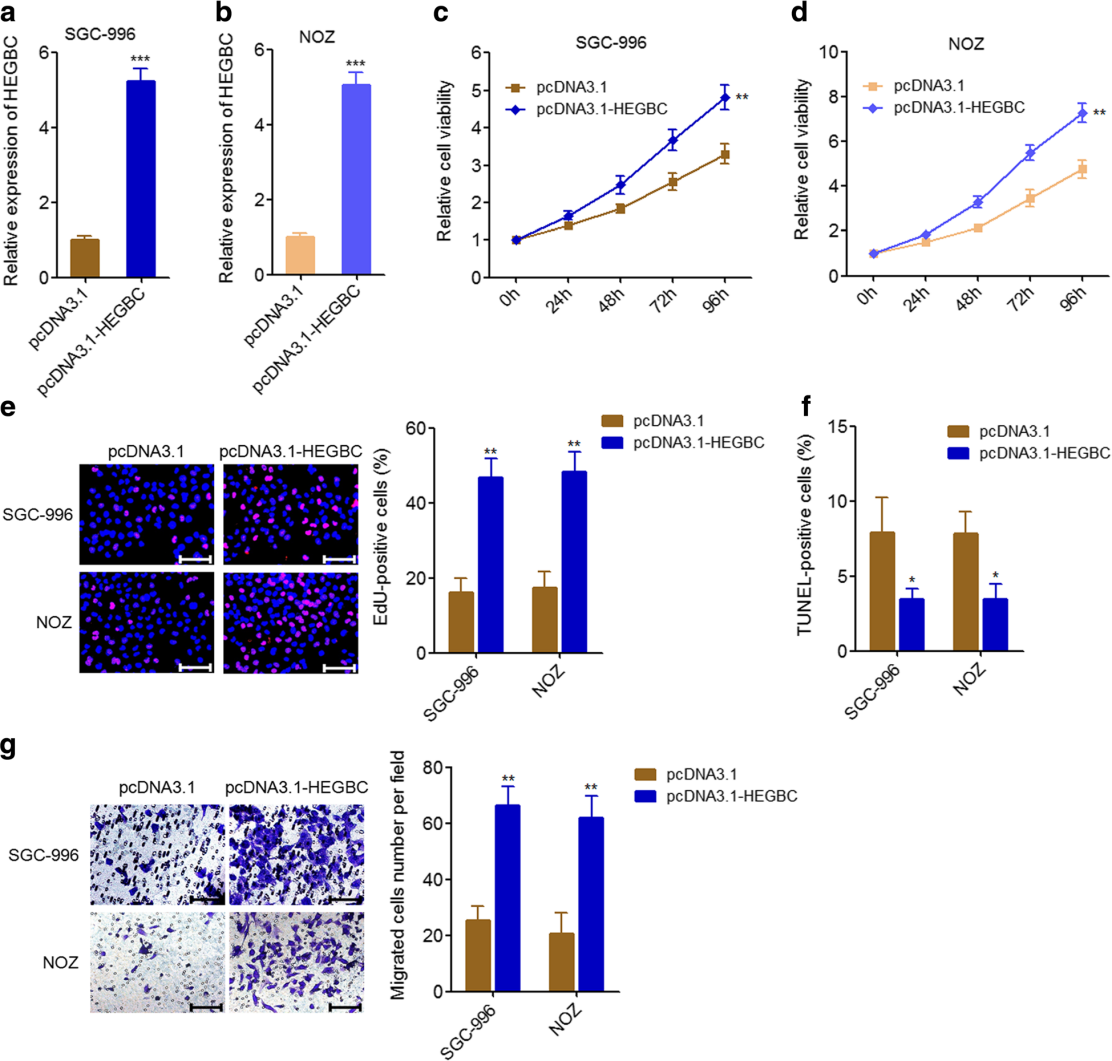
Ectopic expression of HEGBC promoted GBC cell proliferation and migration in vitro. **a** The expression of HEGBC in HEGBC stably overexpressed and control SGC-996 cells was detected using qRT-PCR. **b** The expression of HEGBC in HEGBC stably overexpressed and control NOZ cells was detected using qRT-PCR. **c** Cell proliferation of HEGBC stably overexpressed and control SGC-996 cells was detected using Glo cell viability assay. **d** Cell proliferation of HEGBC stably overexpressed and control NOZ cells was detected using Glo cell viability assay. **e** Cell proliferation of HEGBC stably overexpressed and control SGC-996 and NOZ cells was detected using EdU incorporation assay. The red color represents EdU-positive cells. Scale bars, 100 μm. **f** HEGBC stably overexpressed and control SGC-996 and NOZ cells were treated with 5 ng/mL doxorubicin for 24 h, and then cell apoptosis was detected using TUNEL assay. **g** Cell migration of HEGBC stably overexpressed and control SGC-996 and NOZ cells was detected using transwell assay. Scale bars, 100 μm. Results are shown as mean ± s.d. of 3 independent experiments. *P < 0.05, **P < 0.01, ***P < 0.001 by Student’s t test

To investigate the biological roles of HEGBC in GBC, we constructed HEGBC stably overexpressed SGC-996 and NOZ cells via transfecting HEGBC overexpression plasmid pcDNA3.1-HEGBC (Fig. 2a, b). Glo cell viability assays indicated that ectopic expression of HEGBC significantly promoted cell proliferation of SGC-996 and NOZ cells (Fig. 2c, d). EdU incorporation assays also indicated that ectopic expression of HEGBC markedly promoted cell proliferation of SGC-996 and NOZ cells (Fig. 2e). TUNEL assays indicated that ectopic expression of HEGBC markedly inhibited cell apoptosis of SGC-996 and NOZ cells (Fig. 2f). Transwell assays indicated that ectopic expression of HEGBC significantly promoted cell migration of SGC-996 and NOZ cells (Fig. 2g). Collectively, these results showed that ectopic expression of HEGBC promoted the proliferation and migration of GBC cells in vitro.

### Depletion of HEGBC inhibited the proliferation and migration of GBC cells

**Fig. 3:**
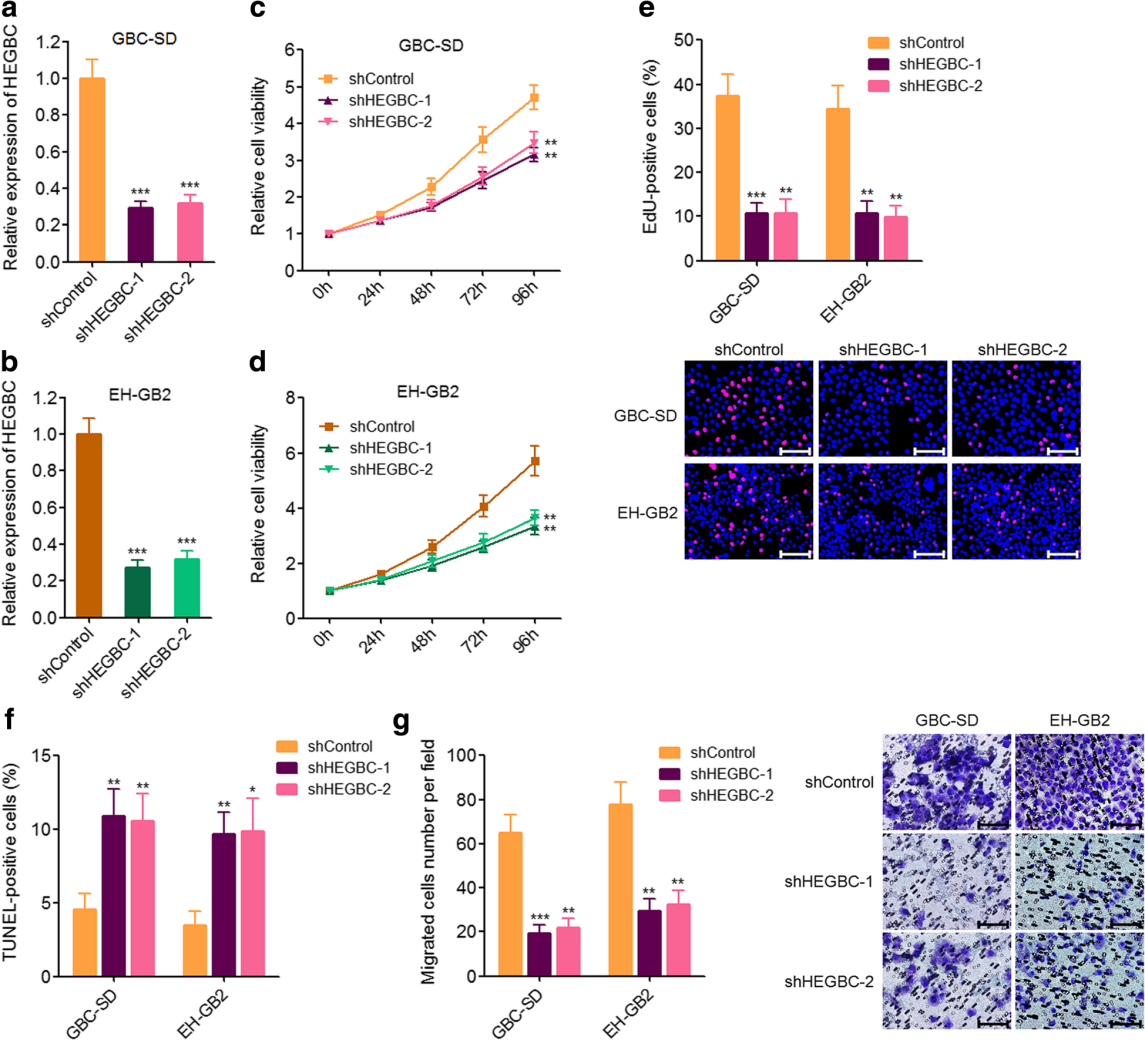
Depletion of HEGBC inhibited GBC cell proliferation and migration in vitro. **a** The expression of HEGBC in HEGBC stably depleted and control GBC-SD cells was detected using qRT-PCR. **b** The expression of HEGBC in HEGBC stably depleted and control EH-GB2 cells was detected using qRT-PCR. **c** Cell proliferation of HEGBC stably depleted and control GBC-SD cells was detected using Glo cell viability assay. **d** Cell proliferation of HEGBC stably depleted and control EH-GB2 cells was detected using Glo cell viability assay. **e** Cell proliferation of HEGBC stably depleted and control GBC-SD and EH-GB2 cells was detected using EdU incorporation assay. The red color represents EdU-positive cells. Scale bars, 100 μm. **f** HEGBC stably depleted and control GBC-SD and EH-GB2 cells were treated with 5 ng/mL doxorubicin for 24 h, and then cell apoptosis was detected using TUNEL assay. **g** Cell migration of HEGBC stably depleted and control GBC-SD and EH-GB2 cells was detected using transwell assay. Scale bars, 100 μm. Results are shown as mean ± s.d. of 3 independent experiments. *P < 0.05, **P < 0.01, ***P < 0.001 by Student’s t test

To completely elucidate the biological roles of HEGBC on GBC cell proliferation and migration, HEGBC stably depleted GBC-SD and EH-GB2 cells were constructed via transfecting two dependent HEGBC specific shRNAs (Fig. 3a, b). Glo cell viability assays indicated that depletion of HEGBC significantly inhibited cell proliferation of GBC-SD and EH-GB2 cells (Fig. 3c, d). EdU incorporation assays also indicated that depletion of HEGBC significantly inhibited cell proliferation of GBC-SD and EH-GB2 cells (Fig. 3e). TUNEL assays indicated that depletion of HEGBC markedly promoted cell apoptosis of GBC-SD and EH-GB2 cells (Fig. 3f). Transwell assays indicated that depletion of HEGBC significantly inhibited cell migration of GBC-SD and EH-GB2 cells (Fig. 3g). Collectively, these results showed that depletion of HEGBC inhibited the proliferation and migration of GBC cells in vitro.

### Ectopic expression of HEGBC promoted tumorigenesis and metastasis of GBC in vivo

**Fig. 4:**
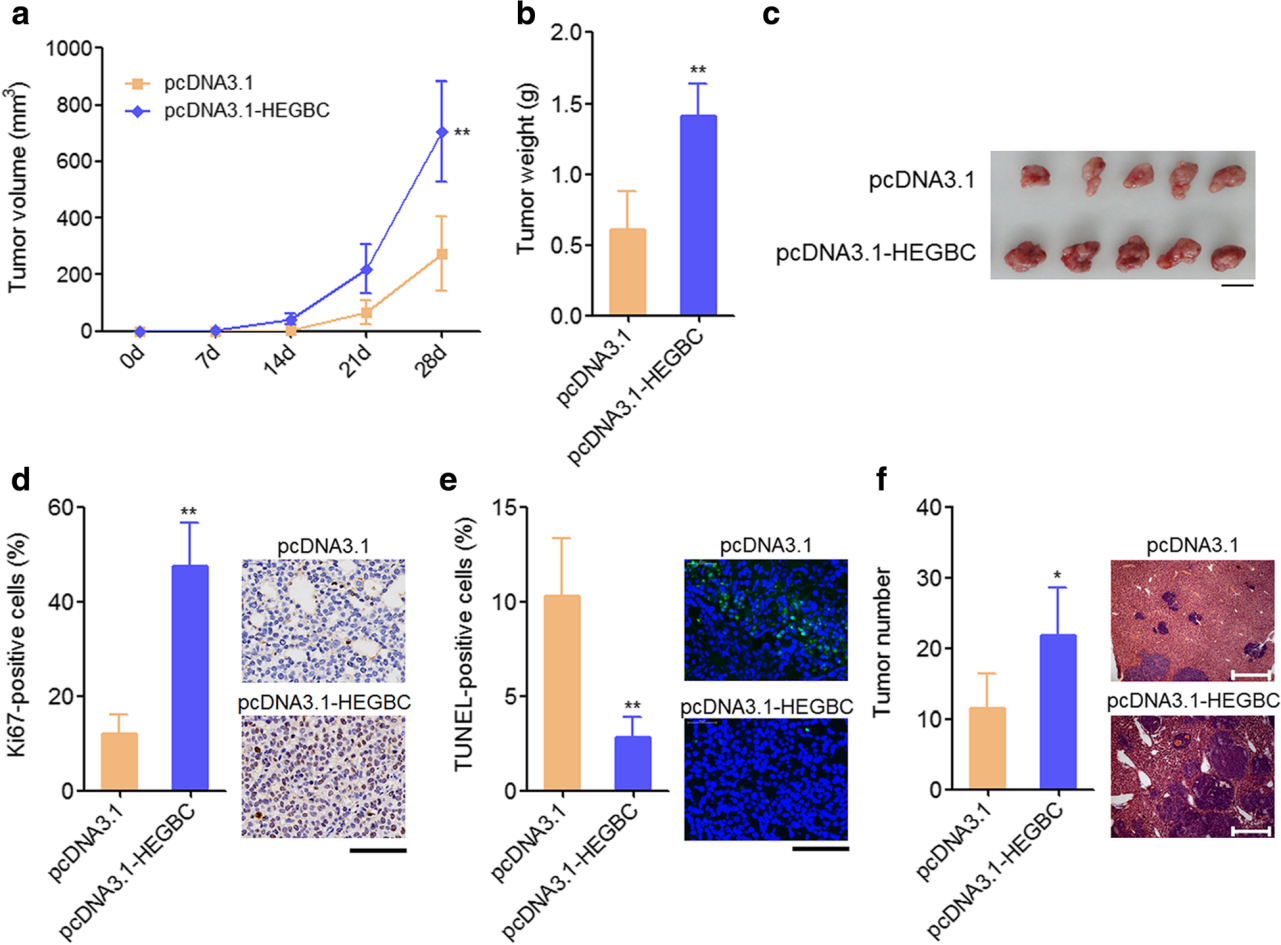
Ectopic expression of HEGBC promoted GBC growth and metastasis in vivo**. a** HEGBC stably overexpressed and control NOZ cells were subcutaneously injected into nude mice. Tumor volumes were detected every 7 days. **b** The subcutaneous tumor weights were detected at the 28th day after injection. **c** Representative images of the subcutaneous tumors formed by HEGBC stably overexpressed and control NOZ cells. Scale bars, 1 cm. **d** Immunohistochemical staining of Ki67 in subcutaneous tumors formed by HEGBC stably overexpressed and control NOZ cells. Scale bars, 100 μm. **e** TUNEL staining of subcutaneous tumors formed by HEGBC stably overexpressed and control NOZ cells. Scale bars, 100 μm. **f** HEGBC stably overexpressed and control NOZ cells were injected through the spleen to establish liver metastasis. The number of liver metastatic foci was detected at the 42th day after intra-splenic injection using HE staining. Scale bars, 400 μm. Results are shown as mean ± s.d. of 5 mice in each group. *P < 0.05, **P < 0.01 by Mann-Whitney test

Next, we investigated the roles of HEGBC on GBC tumorigenesis and metastasis in vivo. HEGBC stably overexpressed and control NOZ cells were subcutaneously injected into nude mice. The results indicated that ectopic expression of HEGBC significantly increased tumor growth rate (Fig. 4a). Also, HEGBC stably overexpressed NOZ cells formed larger subcutaneous tumors than that formed by control NOZ cells (Fig. 4b, c). Proliferation marker Ki67 immunohistochemical staining of the subcutaneous tumors indicated that the tumors formed by HEGBC overexpressed NOZ cells had higher proportion of Ki67-positive cells than that formed by control NOZ cells (Fig. 4d). TUNEL staining of the subcutaneous tumors indicated that the tumors formed by HEGBC overexpressed NOZ cells had lower proportion of apoptotic cells than that formed by control NOZ cells (Fig. 4e). To investigate the roles of HEGBC on GBC metastasis, HEGBC stably overexpressed and control NOZ cells were injected through the spleen to establish liver metastasis model in nude mice. The results indicated that ectopic expression of HEGBC increased liver metastatic foci formed by NOZ cells (Fig. 4f). Collectively, these results showed that ectopic expression of HEGBC promoted GBC tumorigenesis and metastasis in vivo.

### Depletion of HEGBC inhibited tumorigenesis and metastasis of GBC in vivo

**Fig. 5:**
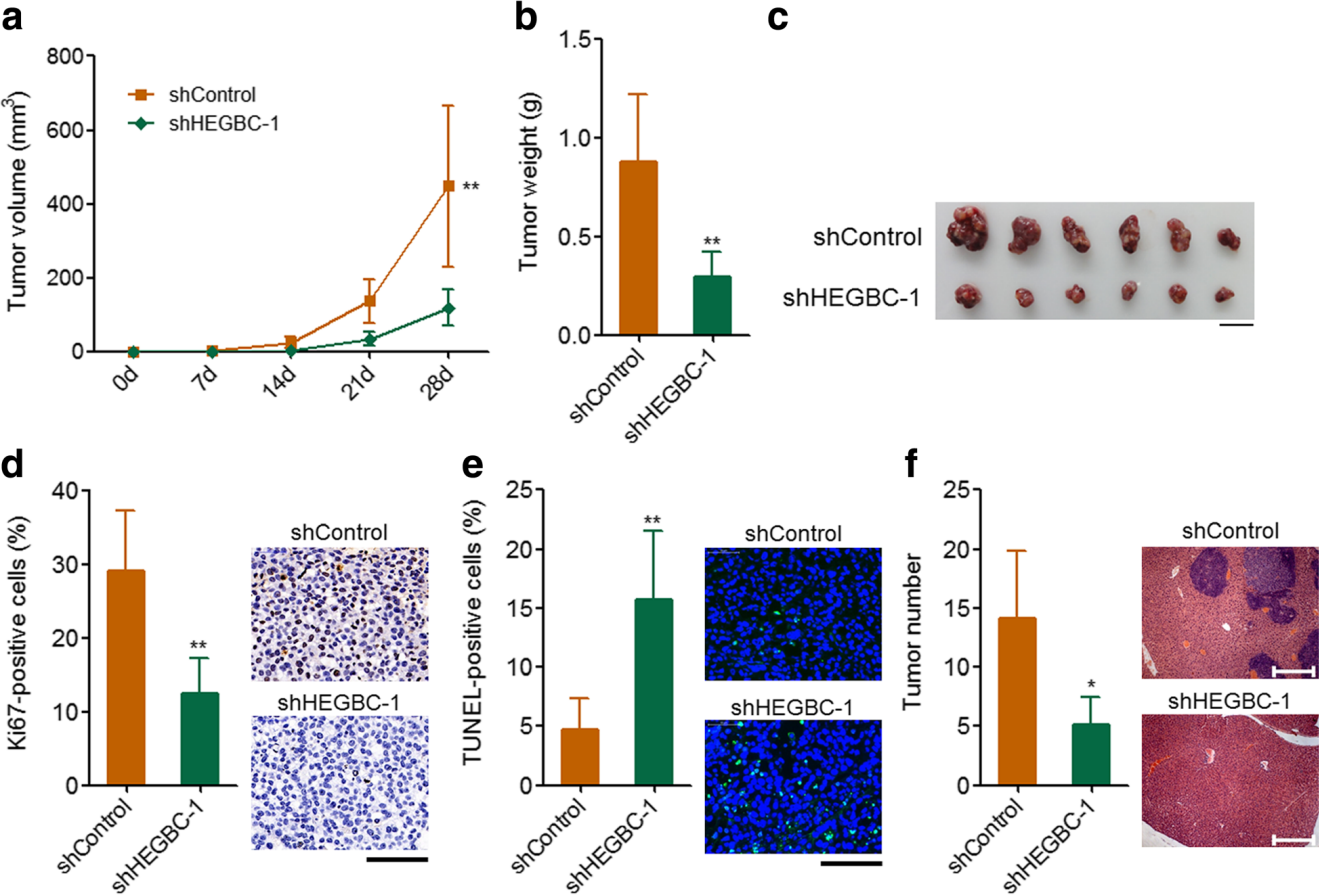
Depletion of HEGBC inhibited GBC growth and metastasis in vivo. **a** HEGBC stably depleted and control EH-GB2 cells were subcutaneously injected into nude mice. Tumor volumes were detected every 7 days. **b** The subcutaneous tumor weights were detected at the 28th day after injection. **c** Representative images of the subcutaneous tumors formed by HEGBC stably depleted and control EH-GB2 cells. Scale bars, 1 cm. **d** Immunohistochemical staining of Ki67 in subcutaneous tumors formed by HEGBC stably depleted and control EH-GB2 cells. Scale bars, 100 μm. **e** TUNEL staining of subcutaneous tumors formed by HEGBC stably depleted and control EH-GB2 cells. Scale bars, 100 μm. **f** HEGBC stably depleted and control EH-GB2 cells were injected through the spleen to establish liver metastasis. The number of liver metastatic foci was detected at the 42th day after intra-splenic injection using HE staining. Scale bars, 400 μm. Results are shown as mean ± s.d. of 6 mice in each group. *P < 0.05, **P < 0.01 by Mann-Whitney test

To completely elucidate the biological roles of HEGBC on GBC tumorigenesis and metastasis in vivo, HEGBC stably depleted EH-GB2 cells were subcutaneously injected into nude mice. The results indicated that depletion of HEGBC significantly decreased tumor growth rate (Fig. 5a). Also, HEGBC stably overexpressed EH-GB2 cells formed larger subcutaneous tumors than that formed by control EH-GB2 cells (Fig. 5b, c). Ki67 immunohistochemical staining of the subcutaneous tumors indicated that the tumors formed by HEGBC depleted EH-GB2 cells had lower proportion of Ki67-positive cells than that formed by control EH-GB2 cells (Fig. 5d). TUNEL staining of the subcutaneous tumors indicated that the tumors formed by HEGBC depleted EH-GB2 cells had higher proportion of apoptotic cells than that formed by control EH-GB2 cells (Fig. 5e). HEGBC stably depleted and control EH-GB2 cells were also injected through the spleen to establish liver metastasis model in nude mice. The results indicated that depletion of HEGBC significantly decreased liver metastatic foci formed by EH-GB2 cells (Fig. 5f). Collectively, these results showed that depletion of HEGBC inhibited GBC tumorigenesis and metastasis in vivo.

### HEGBC bound to *IL-11* promoter and activated IL-11/STAT3 signaling pathway

**Fig. 6:**
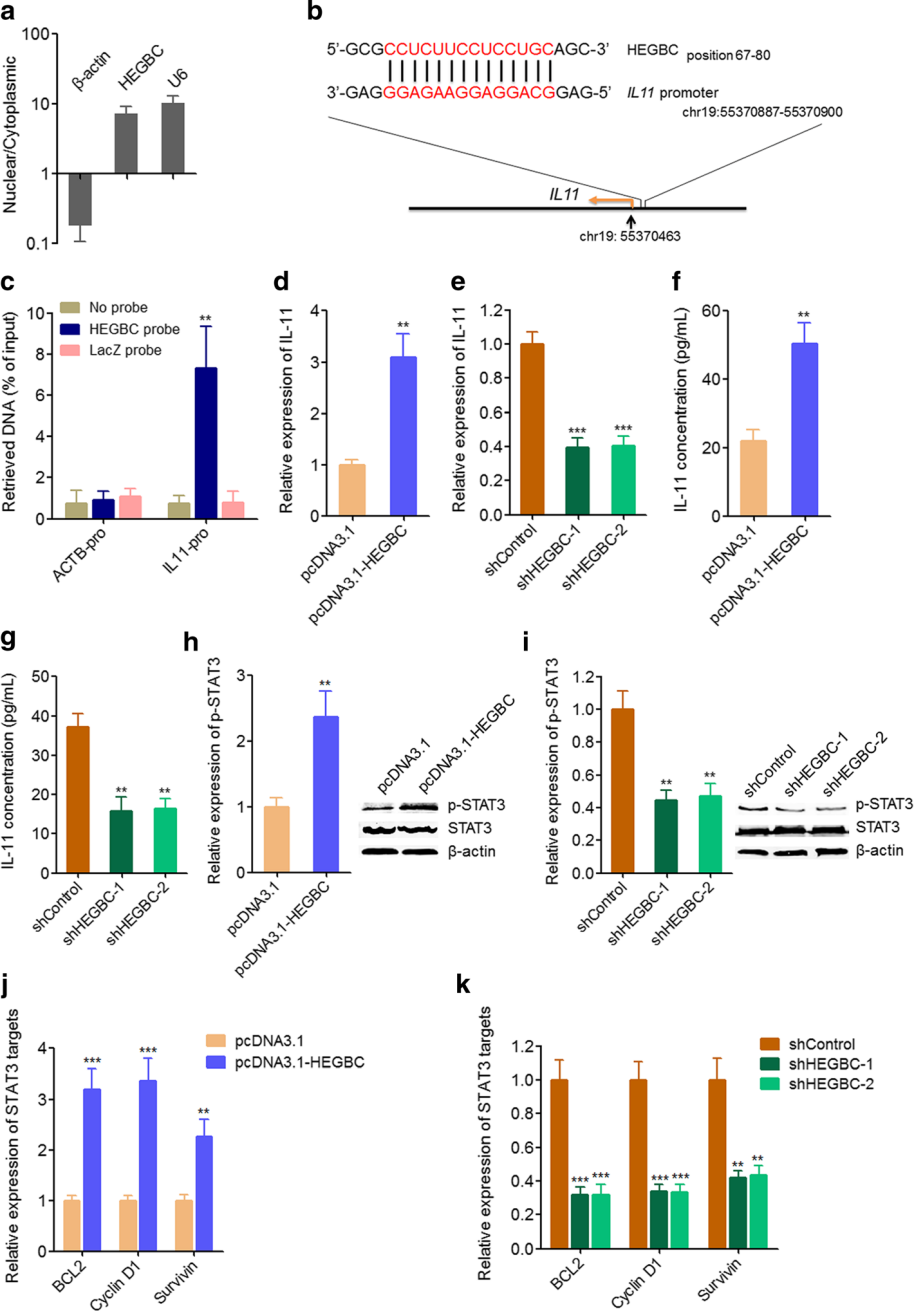
HEGBC bound to the promoter of *IL11* and activated IL-11/STAT3 signaling pathway. **a** The distribution of HEGBC in the cytoplasmic and nuclear fractions of NOZ cells was detected using cytoplasmic and nuclear RNA isolation followed by qRT-PCR. U6 and β-actin were used as nuclear and cytoplasmic controls, respectively. **b** Schematic outline of the predicted binding site for HEGBC on the promoter of *IL11*. **c** ChIRP assay in NOZ cells was performed with antisense probe sets against HEGBC or LacZ (negative control). The bound DNA was detected using qRT-PCR with specific primers against the promoters of *IL11* or *ACTB*. **d** The expression of IL-11 in HEGBC stably overexpressed and control NOZ cells was detected using qRT-PCR. **e** The expression of IL-11 in HEGBC stably depleted and control EH-GB2 cells was detected using qRT-PCR. **f** The concentration of IL11 in the culture medium from HEGBC stably overexpressed and control NOZ cells was detected using ELISA. **g** The concentration of IL11 in the culture medium from HEGBC stably depleted and control EH-GB2 cells was detected using ELISA. **h** STAT3 phosphorylation level in HEGBC stably overexpressed and control NOZ cells was detected using western blot. **i** STAT3 phosphorylation level in HEGBC stably depleted and control EH-GB2 cells was detected using western blot. **j** The expression of the target genes of STAT3 in HEGBC stably overexpressed and control NOZ cells was detected using qRT-PCR. **k** The expression of the target genes of STAT3 in HEGBC stably depleted and control EH-GB2 cells was detected using qRT-PCR. Results are shown as mean ± s.d. of 3 independent experiments. **P < 0.01, ***P < 0.001 by Student’s t test

To investigate the mechanisms mediating the roles of HEGBC in GBC, we first detected the subcellular distribution of HEGBC in GBC cells. Cytoplasmic and nuclear RNA isolation indicted that HEGBC was mainly localized in the nucleus (Fig. 6a). Several nuclear lncRNAs have been reported to directly bind the promoter of target genes and regulate the transcription of target genes [31]. The activation of STAT3 signaling has been reported to be involved in GBC initiation and progression [32, 33]. Therefore, we investigated whether HEGBC regulated the expression of STAT3 signaling inducer via binding their promoters. Intriguingly, using Basic Local Alignment Search Tool (BLAST) from NCBI, we found a HEGBC binding site on the promoter of *IL11* which is well-known to activate STAT3 signaling (Fig. 6b). Moreover, IL-11 was 4.88-fold higher in GBC tissues than corresponding non-tumor tissues from GSE76633 (Additional file 1: Table S1). To investigate whether HEGBC bound to the promoter of *IL11*, ChIRP assays were carried out using antisense probe sets against HEGBC or LacZ (negative control). The results showed that the promoter of *IL11*, but not *ACTB* was markedly enriched in the DNA pulled down by HEGBC antisense probe compared with LacZ antisense probe and no probe (Fig. 6c), suggesting that HEGBC bound to the promoter of *IL11*. To investigate the effects of HEGBC on IL-11 expression, qRT-PCR was carried out on HEGBC stably overexpressed and control NOZ cells. The results showed that ectopic expression of HEGBC up-regulated IL-11 expression (Fig. 6d). Conversely, depletion of HEGBC significantly decreased IL-11 expression in EH-GB2 cells (Fig. 6e). In addition, IL-11 concentrations in the cell supernatants of HEGBC stably overexpressed NOZ cells and HEGBC stably depleted EH-GB2 cells were measured by ELISA assays. The results showed that ectopic expression of HEGBC up-regulated IL-11 concentration, and while depletion of HEGBC decreased IL-11 concentration (Fig. 6f, g). Next, we investigated the effects of HEGBC on STAT3 signaling activation. Western blot assays indicated that ectopic expression of HEGBC up-regulated the phosphorylation level of STAT3, and while depletion of HEGBC decreased the phosphorylation level of STAT3 (Fig. 6h, i). Moreover, ectopic expression of HEGBC up-regulated the expression of the target genes of STAT3, and while depletion of HEGBC decreased the expression of the target genes of STAT3 (Fig. 6j, k). Collectively, these results showed that HEGBC bound to the promoter of *IL11*, up-regulated the expression of IL-11, induced the secretion of IL-11, and activated STAT3 signaling.

### P-STAT3 bound to *HEGBC* promoter and activated HEGBC expression

**Fig. 7:**
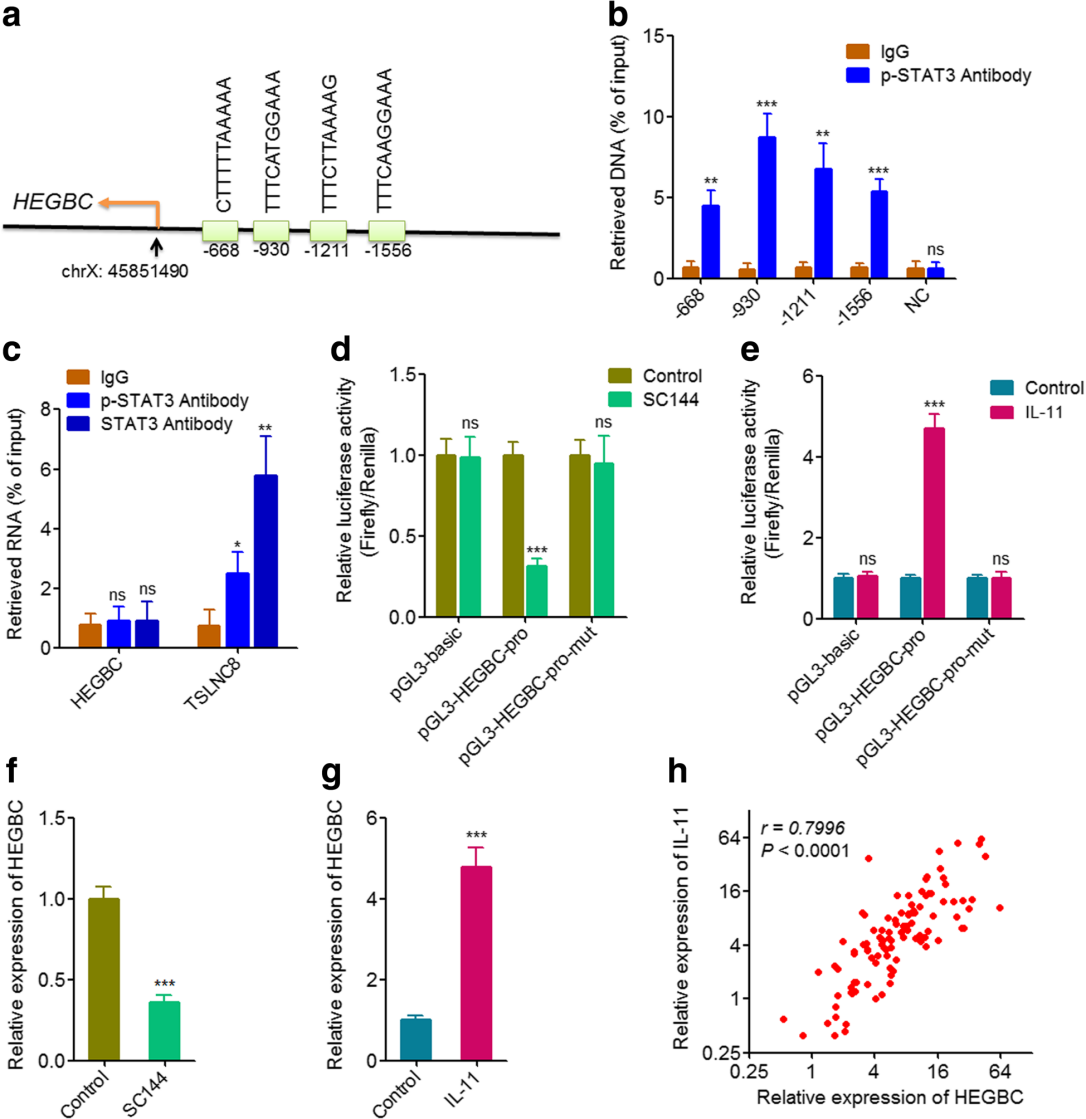
p-STAT3 bound to the promoter of *HEGBC* and activated HEGBC expression. **a** Schematic outline of the predicted binding site for p-STAT3 on the promoter of *HEGBC*. **b** ChIP assay in NOZ cells was performed using p-STAT3 specific antibody or negative control IgG. The bound DNA was detected using qRT-PCR with specific primers against the predicted binding sites on the promoter of *HEGBC* or a distal non-binding site (negative control, NC). **c** RIP assay in NOZ cells was performed using p-STAT3 specific antibody, STAT3 specific antibody, or negative control IgG. The bound RNA was detected using qRT-PCR with specific primers against HEGBC or TSLNC8 (positive control). **d** After co-transfection of renilla luciferase reporter pRL-TK with firefly luciferase reporters containing *HEGBC* promoter, p-STAT3 binding sites mutated *HEGBC* promoter, or nothing into NOZ cells, the NOZ cells were treated with 5 μM p-STAT3 inhibitor SC144 for 72 h. Luciferase activities in these NOZ cells were detected. Data are presented as the relative ratio of firefly luciferase activity to renilla luciferase activity. **e** After co-transfection of renilla luciferase reporter pRL-TK with firefly luciferase reporters containing *HEGBC* promoter, p-STAT3 binding sites mutated *HEGBC* promoter, or nothing into NOZ cells, the NOZ cells were treated with 20 ng/mL IL-11 for 72 h. Luciferase activities in these NOZ cells were detected. Data are presented as the relative ratio of firefly luciferase activity to renilla luciferase activity. **f** The expression of IL-11 in NOZ cells treated with 5 μM SC144 for 72 h was detected using qRT-PCR. **g** The expression of IL-11 in NOZ cells treated with 20 ng/mL IL-11 for 72 h was detected using qRT-PCR. For **b**-**g**, results are shown as mean ± s.d. of 3 independent experiments. ns, not significant, **P < 0.01, ***P < 0.001 by Student’s t test. **h** The correlation between HEGBC and IL-11 expression level in 102 GBC tissues. *r* = 0.7996, *P* < 0.0001 by Pearson’s correlation analysis

Once activated by cytokines and growth factors, such as IL-11, the phosphorylated STAT3 (p-STAT3) translocates to the cell nucleus, binds to the promoters of target genes, and modulates the expression of target genes. To investigate whether HEGBC is the target gene of p-STAT3, we searched the binding sites of p-STAT3 on the promoter of *HEGBC* using JASPAR [34] and identified four potential binding sites (Fig. 7a). ChIP assays were performed on NOZ cells using p-STAT3 specific antibody or control IgG. The results showed that p-STAT3 specifically bound to the four predicted sites on the promoter of *HEGBC*, but not a distal non-binding site (Fig. 7b). Several lncRNAs have been reported to physically bind STAT3, such as TSLNC8 [35]. To investigate whether HEGBC direct binds STAT3, RIP assays were performed using p-STAT3 or STAT3 specific antibody. The results showed that neither p-STAT3 nor STAT3 physically bound HEGBC (Fig. 7c). To investigate whether p-STAT3 regulates promoter activity of *HEGBC*, the promoter of *HEGBC* was subcloned into the luciferase reporter pGL3. After transfection of the constructed luciferase reporter, NOZ cells were treated with 5 μM p-STAT3 inhibitor SC144 for 72 h. Dual luciferase reporter assays showed that inhibiting p-STAT3 decreased the promoter activity of *HEGBC*, but not p-STAT3 binding sites mutated *HEGBC* promoter (Fig. 7d). Conversely, activation of p-STAT3 using IL-11 increased the promoter activity of *HEGBC*, but not p-STAT3 binding sites mutated *HEGBC* promoter (Fig. 7e). Treatment of NOZ cells with SC144 also up-regulated the expression of HEGBC (Fig. 7f), and while treatment of NOZ cells with IL-11 down-regulated the expression of HEGBC (Fig. 7g). The expression of IL-11 in the 102 GBC tissues in Fig. 1a was measured by qRT-PCR. Correlation analysis showed that the expression of HEGBC was significantly positively correlated with the expression of IL-11 in GBC tissues (Fig. 7h), supporting the positive modulation between HEGBC and IL-11. Collectively, these results showed that IL-11/STAT3 signaling activated the expression of HEGBC, and HEGBC and IL-11/STAT3 formed double positive regulatory loop.

### Depletion of IL-11 attenuated the oncogenic roles of HEGBC in GBC cells

**Fig. 8:**
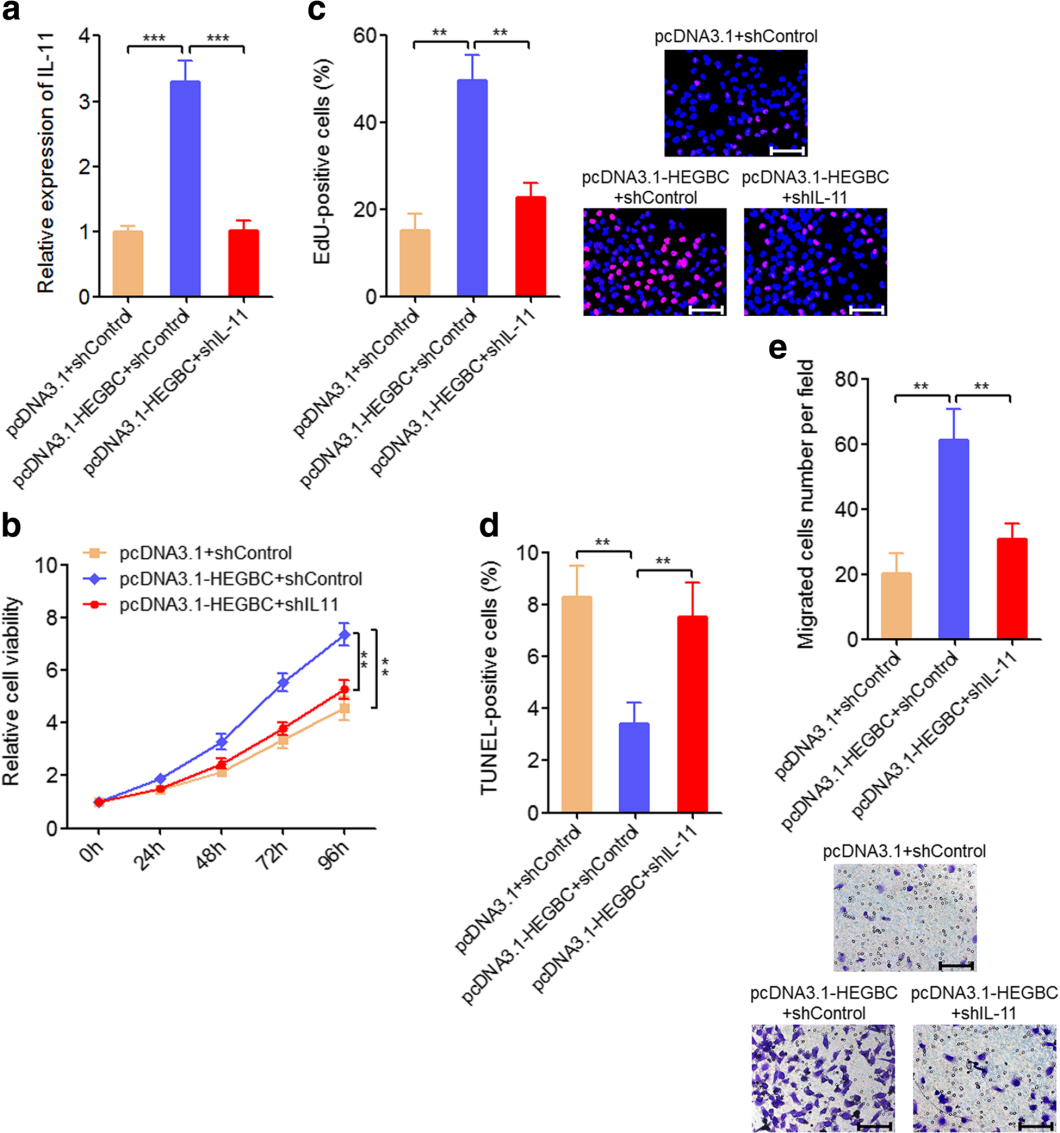
Depletion of IL-11 attenuated the oncogenic roles of HEGBC in GBC cells. **a** The expression of IL-11 in HEGBC stably overexpressed and control NOZ cells transfected with IL-11 specific shRNA was detected using qRT-PCR. **b** Cell proliferation of HEGBC stably overexpressed and control NOZ cells transfected with IL-11 specific shRNA was detected using Glo cell viability assay. **c** Cell proliferation of HEGBC stably overexpressed and control NOZ cells transfected with IL-11 specific shRNA was detected using EdU incorporation assay. The red color represents EdU-positive cells. Scale bars, 100 μm. **d** HEGBC stably overexpressed and control NOZ cells transfected with IL-11 specific shRNA were treated with 5 ng/ml doxorubicin for 24 h, and then cell apoptosis was detected using TUNEL assay. **e** Cell migration of HEGBC stably overexpressed and control NOZ cells transfected with IL-11 specific shRNA was detected using transwell assay. Scale bars, 100 μm. Results are shown as mean ± s.d. of 3 independent experiments. **P < 0.01, ***P < 0.001 by Student’s t test

To investigate whether the activation of IL-11/STAT3 signaling mediates the oncogenic roles of HEGBC in GBC, we knocked-down IL-11 expression in HEGBC stably overexpressed NOZ cells via transfecting IL-11 specific shRNA (Fig. 8a). Glo cell viability assays indicated that depletion of IL-11 attenuated the proliferation promoting roles of HEGBC on NOZ cells (Fig. 8b). EdU incorporation assays also indicated that depletion of IL-11 attenuated the proliferation promoting roles of HEGBC on NOZ cells (Fig. 8c). TUNEL assays indicated that depletion of IL-11 attenuated the apoptosis inhibitory roles of HEGBC on NOZ cells (Fig. 8d). Transwell assays indicated that depletion of IL-11 attenuated the migration promoting roles of HEGBC on NOZ cells (Fig. 8e). Collectively, these results showed that depletion of IL-11 attenuated the oncogenic roles of HEGBC.

## Discussion

Human transcriptome sequencing identified 58,648 lncRNAs, of which 79% were unannotated [36]. Among these lncRNAs, only a few were investigated in cancers. As to GBC, only several lncRNAs were investigated, including SPRY4-IT1 [29], CCAT1 [25], MALAT1 [37], H19 [28], GCASPC [27], UCA1 [38], HOXA-AS2 [26], PAGBC [24], AFAP1-AS1 [39], LET [40], LOC344887 [41], HOTAIR [42], and ROR [43]. Due to the huge number of lncRNAs in human cells, we could not rule out other lncRNAs which may also play critical roles in GBC. Therefore, we re-analyzed the public available microarray data about the differentially expressed lncRNAs in GBC. Intriguingly, we identified a novel lncRNA HEGBC, which is significantly up-regulated in GBC tissues and cell lines, compared with adjacent non-tumor tissues and non-tumorigenic biliary epithelial cell line, respectively. Increased expression of HEGBC is positively correlated with GBC extension, lymph node metastasis, and TNM stages. Furthermore, increased expression of HEGBC predicts poor outcome of GBC patients. Thus, this study identified a novel GBC-associated lncRNA HEGBC which indicates poor prognosis of GBC patients. The expression pattern and clinical significances of HEGBC in other cancers need further investigation.

Subsequently, in vitro functional assays showed that enhanced expression of HEGBC increased cell viabilities, promoted cell proliferation and migration, and inhibited cell apoptosis of GBC cells. Conversely, depletion of HEGBC decreased cell viabilities, inhibited cell proliferation and migration, and induced cell apoptosis of GBC cells. In vivo xenograft assays showed that enhanced expression of HEGBC promoted GBC tumor growth and liver metastasis, and while depletion of HEGBC inhibited GBC tumor growth and liver metastasis. Therefore, our data suggested that HEGBC functions as an oncogene in GBC, and implied that HEGBC may be a potential therapeutic target for GBC. Whether the oncogenic role of HEGBC is GBC specific or tumor general needs further study.

The excessive activation of STAT3 signaling pathway has been found in many cancers, including GBC [44, 45]. In response to stimulation of cytokines or growth factors, STAT3 is phosphorylated by receptor associated kinases, translocates to cell nucleus, and activates the transcription of many target genes that regulating cell viability, apoptosis, and so on. The activated STAT3 has oncogenic roles in many cancers, including GBC [46, 47]. In this study, we found that HEGBC activated STAT3 signaling pathway. Further mechanistic investigations revealed that the nucleus-localized HEGBC directly bound the promoter of *IL11*, which stimulates the activation of STAT3. Via binding the promoter of *IL11*, HEGBC activated the transcription of *IL11*, and further upregulated the expression and secretion of IL-11. The auto-secreted IL-11 further stimulated the activation of STAT3. Intriguingly, except the activation of IL-11/STAT3 signaling by HEGBC, we also found that STAT3 directly bound the promoter of *HEGBC* and activated the transcription of *HEGBC*. Thus, HEGBC/IL-11/STAT3 form a positive regulatory loop in GBC. The double positive regulatory roles amplified the aberrant expressions and roles of the participators. Loss-of-function assays showed that depletion of IL-11 attenuated the oncogenic roles of HEGBC in GBC. In addition, the expression of HEGBC is positively associated with that of IL-11 in GBC tissues. These data supported the positive interaction between HEGBC and IL-11 and suggested that HEGBC exerts its oncogenic roles at least partially via activating IL-11/STAT3 signaling. Although the oncogenic roles of STAT3 are well known, the roles of IL-11 in cancer are relatively unknown. Previous studies have revealed the critical roles of IL-11 in tumorigenesis of liver, colon, and gastric cancers [48, 49]. In this study, we further verified that IL-11 also has critical roles in GBC. Therefore, our data suggest that HEGBC/IL-11/STAT3 regulatory loop may be potential therapeutic targets for GBC.

## Conclusions

In summary, our findings identified a novel lncRNA HEGBC, which is upregulated in GBC and associated with poor prognosis of GBC patients. HEGBC promotes GBC cell proliferation and migration, inhibited GBC cell apoptosis, and promoted tumor growth and metastasis of GBC via forming a positive regulatory loop with IL-11/STAT3. Our data suggested that HEGBC may be a potential prognostic biomarker and therapeutic target for GBC.

## Additional files

Additional file 1: **Table S1.** Top 60 differentially expressed genes in GBC. (DOCX 23 kb)

Additional file 2: **Figure S1.** The full-length sequence of HEGBC. Representative image of PCR products from 5’-RACE and 3’-RACE assays are shown. **Figure S2.** RIP assay in NOZ cells was performed using RPLP0 specific antibody or negative control IgG. The bound RNA was detected using qRT-PCR with specific primers against HEGBC. Results are shown as mean ± s.d. of 3 independent experiments. ns, not significant by Student’s t test. (DOCX 15 kb)

## Abbreviations

ChIP: chromatin immunoprecipitation
ChIRP: chromatin isolation by RNA purification
EdU: ethynyl deoxyuridine
ELISA: enzyme linked immunosorbent assay
GBC: gallbladder cancer
GEO: gene expression omnibus
HEGBC: high expressed in gallbladder cancer
lncRNA: long noncoding RNA
NCBI: national center for biotechnology information
qRT-PCR: quantitative real-time polymerase chain reaction
RACE: rapid amplification of cDNA ends
RIP: RNA immunoprecipitation
TUNEL: terminal deoxynucleotidyl transferase-mediated dUTP nick end labelling

## References

[1] Misra S, Chaturvedi A, Misra NC, Sharma ID (2003). Carcinoma of the gallbladder. Lancet Oncol.

[2] Torre LA, Bray F, Siegel RL, Ferlay J, Lortet-Tieulent J, Jemal A (2015). Global cancer statistics, 2012. CA Cancer J Clin.

[3] Baron TH, Grimm IS, Swanstrom LL (2015). Interventional approaches to gallbladder disease. N Engl J Med.

[4] Butte JM, Matsuo K, Gonen M, D'Angelica MI, Waugh E, Allen PJ, Fong Y, DeMatteo RP, Blumgart L, Endo I (2011). Gallbladder cancer: differences in presentation, surgical treatment, and survival in patients treated at centers in three countries. J Am Coll Surg.

[5] Lee J, Park SH, Chang HM, Kim JS, Choi HJ, Lee MA, Jang JS, Jeung HC, Kang JH, Lee HW (2012). Gemcitabine and oxaliplatin with or without erlotinib in advanced biliary-tract cancer: a multicentre, open-label, randomised, phase 3 study. Lancet Oncol.

[6] Wu XS, Shi LB, Li ML, Ding Q, Weng H, Wu WG, Cao Y, Bao RF, Shu YJ, Ding QC (2014). Evaluation of two inflammation-based prognostic scores in patients with resectable gallbladder carcinoma. Ann Surg Oncol.

[7] Hemminki K, Hemminki A, Forsti A, Sundquist K, Li X (2017). Genetics of gallbladder cancer. Lancet Oncol.

[8] Yan X, Hu Z, Feng Y, Hu X, Yuan J, Zhao SD, Zhang Y, Yang L, Shan W, He Q (2015). Comprehensive genomic characterization of long non-coding RNAs across human cancers. Cancer Cell.

[9] Batista PJ, Chang HY (2013). Long noncoding RNAs: cellular address codes in development and disease. Cell.

[10] Ponting CP, Oliver PL, Reik W (2009). Evolution and functions of long noncoding RNAs. Cell.

[11] Schmitt AM, Chang HY (2016). Long noncoding RNAs in Cancer pathways. Cancer Cell.

[12] Leucci E, Vendramin R, Spinazzi M, Laurette P, Fiers M, Wouters J, Radaelli E, Eyckerman S, Leonelli C, Vanderheyden K (2016). Melanoma addiction to the long non-coding RNA SAMMSON. Nature.

[13] Yuan JH, Liu XN, Wang TT, Pan W, Tao QF, Zhou WP, Wang F, Sun SH (2017). The MBNL3 splicing factor promotes hepatocellular carcinoma by increasing PXN expression through the alternative splicing of lncRNA-PXN-AS1. Nat Cell Biol.

[14] Xu D, Yang F, Yuan JH, Zhang L, Bi HS, Zhou CC, Liu F, Wang F, Sun SH (2013). Long noncoding RNAs associated with liver regeneration 1 accelerates hepatocyte proliferation during liver regeneration by activating Wnt/beta-catenin signaling. Hepatology.

[15] Zhang C, Yuan J, Hu H, Chen W, Liu M, Zhang J, Sun S, Guo Z (2017). Long non-coding RNA CHCHD4P4 promotes epithelial-mesenchymal transition and inhibits cell proliferation in calcium oxalate-induced kidney damage. Braz J Med Biol Res.

[16] Lin A, Hu Q, Li C, Xing Z, Ma G, Wang C, Li J, Ye Y, Yao J, Liang K (2017). The LINK-A lncRNA interacts with PtdIns (3,4,5) P3 to hyperactivate AKT and confer resistance to AKT inhibitors. Nat Cell Biol.

[17] Grelet S, Link LA, Howley B, Obellianne C, Palanisamy V, Gangaraju VK, Diehl JA, Howe PH (2017). A regulated PNUTS mRNA to lncRNA splice switch mediates EMT and tumour progression. Nat Cell Biol.

[18] Reis EM, Verjovski-Almeida S (2012). Perspectives of long non-coding RNAs in Cancer diagnostics. Front Genet.

[19] Zhang L, Yang F, Yuan JH, Yuan SX, Zhou WP, Huo XS, Xu D, Bi HS, Wang F, Sun SH (2013). Epigenetic activation of the MiR-200 family contributes to H19-mediated metastasis suppression in hepatocellular carcinoma. Carcinogenesis.

[20] Zhu XT, Yuan JH, Zhu TT, Li YY, Cheng XY (2016). Long noncoding RNA glypican 3 (GPC3) antisense transcript 1 promotes hepatocellular carcinoma progression via epigenetically activating GPC3. FEBS J.

[21] Deng L, Yang SB, Xu FF, Zhang JH (2015). Long noncoding RNA CCAT1 promotes hepatocellular carcinoma progression by functioning as let-7 sponge. J Exp Clin Cancer Res.

[22] Lin A, Li C, Xing Z, Hu Q, Liang K, Han L, Wang C, Hawke DH, Wang S, Zhang Y (2016). The LINK-A lncRNA activates normoxic HIF1alpha signalling in triple-negative breast cancer. Nat Cell Biol.

[23] Li JK, Chen C, Liu JY, Shi JZ, Liu SP, Liu B, Wu DS, Fang ZY, Bao Y, Jiang MM (2017). Long noncoding RNA MRCCAT1 promotes metastasis of clear cell renal cell carcinoma via inhibiting NPR3 and activating p38-MAPK signaling. Mol Cancer.

[24] Wu XS, Wang F, Li HF, Hu YP, Jiang L, Zhang F, Li ML, Wang XA, Jin YP, Zhang YJ (2017). LncRNA-PAGBC acts as a microRNA sponge and promotes gallbladder tumorigenesis. EMBO Rep.

[25] Ma MZ, Chu BF, Zhang Y, Weng MZ, Qin YY, Gong W, Quan ZW (2015). Long non-coding RNA CCAT1 promotes gallbladder cancer development via negative modulation of miRNA-218-5p. Cell Death Dis.

[26] Zhang P, Cao P, Zhu X, Pan M, Zhong K, He R, Li Y, Jiao X, Gao Y (2017). Upregulation of long non-coding RNA HOXA-AS2 promotes proliferation and induces epithelial-mesenchymal transition in gallbladder carcinoma. Oncotarget.

[27] Ma MZ, Zhang Y, Weng MZ, Wang SH, Hu Y, Hou ZY, Qin YY, Gong W, Zhang YJ, Kong X (2016). Long noncoding RNA GCASPC, a target of miR-17-3p, negatively regulates pyruvate carboxylase-dependent cell proliferation in gallbladder Cancer. Cancer Res.

[28] Wang SH, Ma F, Tang ZH, Wu XC, Cai Q, Zhang MD, Weng MZ, Zhou D, Wang JD, Quan ZW (2016). Long non-coding RNA H19 regulates FOXM1 expression by competitively binding endogenous miR-342-3p in gallbladder cancer. J Exp Clin Cancer Res.

[29] Yang L, Cheng X, Ge N, Guo W, Feng F, Wan F (2017). Long non-coding RNA SPRY4-IT1 promotes gallbladder carcinoma progression. Oncotarget.

[30] Wang JH, Li LF, Yu Y, Li B, Jin HJ, Shen DH, Li J, Jiang XQ, Qian QJ (2012). Establishment and characterization of a cell line, EH-GB2, derived from hepatic metastasis of gallbladder cancer. Oncol Rep.

[31] Wang X, Sun W, Shen W, Xia M, Chen C, Xiang D, Ning B, Cui X, Li H, Li X (2016). Long non-coding RNA DILC regulates liver cancer stem cells via IL-6/STAT3 axis. J Hepatol.

[32] Fu LX, Lian QW, Pan JD, Xu ZL, Zhou TM, Ye B (2017). JAK2 tyrosine kinase inhibitor AG490 suppresses cell growth and invasion of gallbladder cancer cells via inhibition of JAK2/STAT3 signaling. J Biol Regul Homeost Agents.

[33] Enyu L, Na W, Chuanzong Z, Ben W, Xiaojuan W, Yan W, Zequn L, Jianguo H, Jiayong W, Benjia L (2017). The clinical significance and underlying correlation of pStat-3 and integrin alphavbeta6 expression in gallbladder cancer. Oncotarget.

[34] Sandelin A, Alkema W, Engstrom P, Wasserman WW, Lenhard B (2004). JASPAR: an open-access database for eukaryotic transcription factor binding profiles. Nucleic Acids Res.

[35] Zhang J, Li Z, Liu L, Wang Q, Li S, Chen D, Hu Z, Yu T, Ding J, Li J (2018). Long noncoding RNA TSLNC8 is a tumor suppressor that inactivates the interleukin-6/STAT3 signaling pathway. Hepatology.

[36] Iyer MK, Niknafs YS, Malik R, Singhal U, Sahu A, Hosono Y, Barrette TR, Prensner JR, Evans JR, Zhao S (2015). The landscape of long noncoding RNAs in the human transcriptome. Nat Genet.

[37] Sun KK, Hu PP, Xu F (2018). Prognostic significance of long non-coding RNA MALAT1 for predicting the recurrence and metastasis of gallbladder cancer and its effect on cell proliferation, migration, invasion, and apoptosis. J Cell Biochem.

[38] Cai Q, Jin L, Wang S, Zhou D, Wang J, Tang Z, Quan Z (2017). Long non-coding RNA UCA1 promotes gallbladder cancer progression by epigenetically repressing p21 and E-cadherin expression. Oncotarget.

[39] Ma F, Wang SH, Cai Q, Zhang MD, Yang Y, Ding J (2016). Overexpression of LncRNA AFAP1-AS1 predicts poor prognosis and promotes cells proliferation and invasion in gallbladder cancer. Biomed Pharmacother.

[40] Wang PL, Liu B, Xia Y, Pan CF, Ma T, Chen YJ (2016). Long non-coding RNA-low expression in tumor inhibits the invasion and metastasis of esophageal squamous cell carcinoma by regulating p53 expression. Mol Med Rep.

[41] Wu XC, Wang SH, Ou HH, Zhu B, Zhu Y, Zhang Q, Yang Y, Li H (2017). The NmrA-like family domain containing 1 pseudogene Loc344887 is amplified in gallbladder cancer and promotes epithelial-mesenchymal transition. Chem Biol Drug Des.

[42] Ma MZ, Li CX, Zhang Y, Weng MZ, Zhang MD, Qin YY, Gong W, Quan ZW (2014). Long non-coding RNA HOTAIR, a c-Myc activated driver of malignancy, negatively regulates miRNA-130a in gallbladder cancer. Mol Cancer.

[43] Wang SH, Zhang MD, Wu XC, Weng MZ, Zhou D, Quan ZW (2016). Overexpression of LncRNA-ROR predicts a poor outcome in gallbladder cancer patients and promotes the tumor cells proliferation, migration, and invasion. Tumour Biol.

[44] Steder M, Alla V, Meier C, Spitschak A, Pahnke J, Furst K, Kowtharapu BS, Engelmann D, Petigk J, Egberts F (2013). DNp73 exerts function in metastasis initiation by disconnecting the inhibitory role of EPLIN on IGF1R-AKT/STAT3 signaling. Cancer Cell.

[45] Kesselring R, Glaesner J, Hiergeist A, Naschberger E, Neumann H, Brunner SM, Wege AK, Seebauer C, Kohl G, Merkl S (2016). IRAK-M expression in tumor cells supports colorectal Cancer progression through reduction of antimicrobial defense and stabilization of STAT3. Cancer Cell.

[46] David D, Rajappan LM, Balachandran K, Thulaseedharan JV, Nair AS, Pillai RM (2011). Prognostic significance of STAT3 and phosphorylated STAT3 in human soft tissue tumors - a clinicopathological analysis. J Exp Clin Cancer Res.

[47] Granato M, Gilardini Montani MS, Santarelli R, D'Orazi G, Faggioni A, Cirone M (2017). Apigenin, by activating p53 and inhibiting STAT3, modulates the balance between pro-apoptotic and pro-survival pathways to induce PEL cell death. J Exp Clin Cancer Res.

[48] Putoczki TL, Thiem S, Loving A, Busuttil RA, Wilson NJ, Ziegler PK, Nguyen PM, Preaudet A, Farid R, Edwards KM (2013). Interleukin-11 is the dominant IL-6 family cytokine during gastrointestinal tumorigenesis and can be targeted therapeutically. Cancer Cell.

[49] Yuan JH, Yang F, Wang F, Ma JZ, Guo YJ, Tao QF, Liu F, Pan W, Wang TT, Zhou CC (2014). A long noncoding RNA activated by TGF-beta promotes the invasion-metastasis cascade in hepatocellular carcinoma. Cancer Cell.

