# Supplementary material for: Long noncoding RNA HEGBC promotes tumorigenesis and metastasis of gallbladder cancer via forming a positive feedback loop with IL-11/STAT3 signaling pathway
Source: J Exp Clin Cancer Res. 2018 Aug 7;37:186. doi: 10.1186/s13046-018-0847-7 (PMC6081844; doi:10.1186/s13046-018-0847-7)
Supplement: Supplementary file 1 — Table S1. Top 60 differentially expressed genes in GBC. (DOCX 23 kb) [file 13046_2018_847_MOESM1_ESM.docx]

**Additional file 1: Table S1** Top 60 differentially expressed genes in GBC

| **Agilent Probe Name** | **Gene Name** | **Fold change (GBC tissue/paired adjacent normal gallbladder tissue )** | | | | | | | | | **Average fold change** |
| --- | --- | --- | --- | --- | --- | --- | --- | --- | --- | --- | --- |
|  |  | Patient 1 | Patient 2 | Patient 3 | Patient 4 | Patient 5 | Patient 6 | Patient 7 | Patient 8 | Patient 9 |  |
| agiseq50260 | CKMT1A | 10.23948 | 7.891517 | 7.51486 | 7.067685 | 8.408414 | 11.25189 | 5.937954 | 10.68437 | 12.4984 | 9.054953 |
| agiseq26628 | PITX1 | 8.115151 | 12.7092 | 8.272037 | 6.529296 | 12.34873 | 10.67263 | 3.845164 | 6.955937 | 8.446836 | 8.654998 |
| CUST_3049_PI428631609 | ENST00000423943 (lncRNA-PAGBC) | 2.995515 | 7.956089 | 9.789543 | 2.400394 | 14.29763 | 7.408141 | 11.92871 | 6.362888 | 11.82888 | 8.329754 |
| pl111726 | uc003swh (IGF2BP3) | 9.808951 | 11.35844 | 8.444084 | 5.393846 | 8.529306 | 6.729354 | 10.24165 | 9.114065 | 4.500927 | 8.235625 |
| agiseq33267 | FGFBP1 | 12.64728 | 2.771518 | 10.79599 | 8.137366 | 4.752131 | 11.78621 | 12.01211 | 3.179018 | 6.954235 | 8.115094 |
| agiseq14858 | GJB4 | 4.122294 | 8.976699 | 9.144297 | 4.362487 | 11.62083 | 9.246264 | 11.45955 | 10.88414 | 2.895723 | 8.079143 |
| pl058877 | NR_038849.1 (lncRNA-PAGBC) | 8.779387 | 4.931904 | 7.542328 | 3.184539 | 10.69469 | 11.2396 | 8.824062 | 3.708759 | 10.9521 | 7.76193 |
| agiseq48224 | SGPP2 | 9.101651 | 7.620683 | 4.827089 | 3.988222 | 8.263832 | 7.558494 | 8.433476 | 4.931715 | 10.32924 | 7.228266 |
| agiseq25538 | CLDN18 | 2.580244 | 7.398934 | 10.23733 | 7.983568 | 3.063537 | 11.89946 | 1.757582 | 12.45474 | 4.003986 | 6.819932 |
| agiseq15279 | GJB5 | 7.665201 | 10.16313 | 3.05945 | 2.462455 | 12.04539 | 5.660246 | 8.375716 | 9.928435 | 1.827007 | 6.798559 |
| pl003784 | exon1991 | 7.808157 | 8.056075 | 6.892167 | 2.600221 | 6.863597 | 7.574779 | 6.388709 | 5.672937 | 8.395861 | 6.694723 |
| agiseq30303 | FUT2 | 6.536999 | 4.040069 | 6.236267 | 2.961293 | 7.131853 | 7.670955 | 6.949762 | 7.277611 | 10.1676 | 6.552489 |
| agiseq8613 | NR_038835 (MNX1-AS1) | 9.740557 | 3.630376 | 7.432869 | 3.709058 | 4.325457 | 8.96822 | 3.273206 | 4.231273 | 13.64267 | 6.550409 |
| agiseq48832 | EGFL6 | 9.617347 | 7.588051 | 10.53074 | 7.509167 | 2.269551 | 7.352095 | 3.276782 | 7.956379 | 2.826373 | 6.547387 |
| agiseq38492 | MNX1 | 3.815439 | 6.149707 | 8.692751 | 1.972168 | 7.841848 | 12.11773 | 3.075103 | 9.166904 | 5.699222 | 6.50343 |
| pl010628 | uc003iiz (INPP4B) | 6.695607 | 4.481814 | 7.651542 | 2.921518 | 1.982931 | 7.636495 | 9.945382 | 6.179862 | 8.383359 | 6.208723 |
| pl114925 | NR_037583.1 (LEMD1) | 7.733603 | 4.77968 | 2.009833 | 1.917646 | 9.27851 | 7.481927 | 9.184266 | 5.996399 | 7.22968 | 6.179061 |
| agiseq46397 | KRT14 | 4.759276 | 8.204237 | 5.544528 | 6.665903 | 3.791568 | 12.74465 | 6.006871 | 2.469855 | 5.00731 | 6.132689 |
| pl058949 | uc002uku (HAGLR) | 3.565545 | 4.878975 | 7.01041 | 7.198846 | 8.32107 | 5.244141 | 5.775805 | 2.094755 | 10.6079 | 6.077494 |
| agiseq17853 | SHCBP1 | 9.158985 | 9.12793 | 5.227836 | 7.974995 | 2.218188 | 7.272824 | 1.82673 | 9.027603 | 2.200892 | 6.003998 |
| agiseq2807 | NPTX1 | 10.51857 | 2.484961 | 2.26132 | 3.371183 | 8.59231 | 8.6727 | 5.964525 | 6.866645 | 4.719161 | 5.939041 |
| pl069343 | ENST00000532855 (MMP12) | 3.244088 | 11.46667 | 6.818486 | 3.893563 | 8.242649 | 5.809969 | 3.338526 | 3.921194 | 6.556462 | 5.92129 |
| agiseq49610 | VGF | 10.49067 | 4.122191 | 7.468776 | 5.104967 | 5.221991 | 7.121203 | 2.543539 | 6.024787 | 4.86316 | 5.884588 |
| pl072319 | AL137342 (UGT8) | 3.417668 | 7.239462 | 5.067568 | 4.93495 | 5.171371 | 2.258428 | 4.400635 | 6.569373 | 13.80574 | 5.873911 |
| agiseq27285 | FRMD5 | 6.677411 | 4.385057 | 8.354007 | 2.940184 | 2.590625 | 7.404414 | 7.523721 | 2.595099 | 8.715932 | 5.687383 |
| **CUST_12783_PI428631609** | **ENST00000414772** | **8.828757** | **7.661031** | **8.705558** | **4.873175** | **2.706307** | **8.86893** | **1.886739** | **6.09863** | **1.543915** | **5.685894** |
| agiseq42469 | C15orf42 | 6.780408 | 7.924471 | 5.63321 | 3.561622 | 4.057728 | 6.729906 | 5.351888 | 3.391435 | 7.741235 | 5.685767 |
| agiseq23008 | ANLN | 10.6884 | 6.574375 | 10.42506 | 3.072485 | 2.386658 | 8.537953 | 1.710494 | 4.138905 | 2.890129 | 5.602717 |
| agiseq422 | GPRC5A | 2.193906 | 2.477468 | 12.4048 | 8.651512 | 2.120982 | 3.527752 | 4.433175 | 1.58232 | 12.9592 | 5.594568 |
| agiseq21070 | LEMD1 | 5.516885 | 1.91459 | 4.917589 | 4.890381 | 7.723484 | 6.646495 | 8.84849 | 4.108259 | 5.415276 | 5.553494 |
| agiseq21716 | ADAMDEC1 | 3.934786 | 8.261534 | 4.672984 | 5.045469 | 6.495222 | 2.953093 | 3.961777 | 9.125466 | 5.422671 | 5.541445 |
| agiseq42607 | NR_026975 | 2.629745 | 4.435439 | 6.63791 | 3.656294 | 4.047363 | 9.094497 | 7.924264 | 8.045961 | 3.365294 | 5.537419 |
| pl053909 | uc010hpe | 5.379401 | 4.720423 | 4.569648 | 6.096449 | 6.210362 | 4.143813 | 8.193799 | 6.810847 | 3.349673 | 5.497157 |
| agiseq20105 | CENPA | 9.600782 | 3.967273 | 8.128427 | 5.434977 | 3.669357 | 8.114478 | 1.753901 | 3.887457 | 4.518379 | 5.452781 |
| pl003939 | uc003swh | 9.858051 | 3.197785 | 8.344963 | 1.740781 | 7.256217 | 1.913678 | 10.94241 | 2.651629 | 2.973139 | 5.430961 |
| agiseq24062 | E2F7 | 5.795208 | 8.216955 | 1.517415 | 4.309707 | 7.327447 | 3.607851 | 5.567335 | 5.334779 | 7.08416 | 5.417873 |
| CUST_11317_PI428631609 | ENST00000526061 | 3.036774 | 3.440819 | 8.272535 | 1.828546 | 5.981547 | 3.935343 | 4.955957 | 6.267473 | 10.24505 | 5.329338 |
| CUST_10769_PI428631609 | ENST00000545819 | 5.282427 | 4.788637 | 4.771033 | 4.147536 | 4.674434 | 6.612198 | 6.430051 | 4.358956 | 6.273551 | 5.259869 |
| agiseq16556 | CALB2 | 7.305916 | 4.467661 | 4.521569 | 7.885067 | 2.037236 | 6.34618 | 8.817336 | 1.763219 | 4.067329 | 5.245724 |
| agiseq32346 | C17orf99 | 8.319203 | 5.729809 | 5.088761 | 4.823926 | 4.123911 | 2.106315 | 6.423052 | 6.185796 | 4.385174 | 5.242883 |
| pl033485 | uc010hpe | 3.991424 | 5.286986 | 5.297785 | 5.777785 | 6.169539 | 4.287745 | 6.791324 | 6.631569 | 2.620949 | 5.206123 |
| agiseq49164 | KCTD16 | 2.392197 | 3.291574 | 7.453677 | 5.961763 | 3.416979 | 7.028922 | 6.182029 | 5.013641 | 5.708511 | 5.161032 |
| agiseq25608 | FAM81A | 5.178812 | 3.97441 | 4.710726 | 3.661858 | 8.333957 | 4.927427 | 4.143301 | 7.460394 | 3.908573 | 5.144384 |
| pl039657 | NR_037892.1 | 2.468123 | 1.90881 | 4.454089 | 4.12632 | 8.207294 | 5.835263 | 5.091051 | 5.741044 | 8.385336 | 5.135259 |
| pl062212 | uc003swh | 5.525047 | 7.587914 | 4.581165 | 2.384003 | 3.094338 | 2.445146 | 6.574465 | 5.994205 | 7.972034 | 5.128702 |
| agiseq13383 | MGC4294 | 2.791964 | 1.927312 | 7.753086 | 6.024651 | 5.084881 | 7.467763 | 8.344146 | 4.521397 | 1.808412 | 5.080401 |
| agiseq11475 | SLC7A11 | 3.18628 | 8.169391 | 7.022337 | 3.985506 | 3.798763 | 2.302215 | 2.905583 | 6.864146 | 7.451804 | 5.076225 |
| agiseq43681 | NMU | 4.56344 | 4.275834 | 7.348937 | 3.513379 | 5.604472 | 3.722438 | 5.495186 | 2.686638 | 8.471532 | 5.075762 |
| CUST_13684_PI428631609 | ENST00000523427 | 3.927546 | 4.75116 | 4.374273 | 3.714343 | 7.43104 | 4.673391 | 2.444487 | 9.285561 | 4.869366 | 5.052352 |
| agiseq12938 | NR_038849 | 2.805667 | 3.652617 | 6.088473 | 2.20006 | 6.536104 | 10.44705 | 2.726703 | 2.991009 | 7.693262 | 5.01566 |
| pl017449 | NR_036685.1 | 3.995983 | 5.084085 | 9.094998 | 3.990948 | 1.819865 | 7.941215 | 2.287177 | 6.820075 | 3.825395 | 4.984416 |
| agiseq39596 | BCAS1 | 3.101951 | 2.07507 | 3.594529 | 2.310571 | 2.080266 | 11.57703 | 10.6329 | 6.142915 | 3.314817 | 4.981116 |
| agiseq23308 | SPOCD1 | 2.562261 | 8.179697 | 8.994241 | 6.530297 | 3.396155 | 5.821397 | 3.288347 | 3.091469 | 2.833696 | 4.966396 |
| pl048629 | CAR | 6.899057 | 6.143471 | 8.076371 | 2.873231 | 2.698121 | 1.902361 | 6.269984 | 4.997694 | 4.73182 | 4.954679 |
| pl037831 | uc010bih | 5.876252 | 6.41486 | 3.659273 | 3.84645 | 7.241239 | 1.784625 | 3.320639 | 6.225425 | 6.069542 | 4.937589 |
| pl058036 | HMlincRNA965 | 1.640357 | 6.804357 | 7.267349 | 2.902381 | 5.579678 | 4.357644 | 2.704772 | 2.647673 | 10.42464 | 4.925428 |
| pl052191 | AK021730 | 5.729211 | 5.69659 | 7.628325 | 4.890823 | 3.918551 | 7.840347 | 2.888836 | 3.26182 | 2.365594 | 4.913344 |
| pl068555 | uc001kxk | 4.052707 | 5.187158 | 5.616911 | 3.730923 | 2.940924 | 5.383662 | 5.912305 | 3.239903 | 8.064668 | 4.90324 |
| agiseq16274 | PODNL1 | 5.66974 | 6.029199 | 6.765416 | 4.881524 | 2.264233 | 6.896764 | 3.140118 | 6.220193 | 2.064652 | 4.881315 |
| **agiseq36934** | **IL11** | **6.994039** | **10.39277** | **3.110898** | **2.359449** | **7.31269** | **5.436508** | **2.165029** | **4.313935** | **1.845681** | **4.881222** |
