# Supplementary material for: Long noncoding RNA HEGBC promotes tumorigenesis and metastasis of gallbladder cancer via forming a positive feedback loop with IL-11/STAT3 signaling pathway
Source: J Exp Clin Cancer Res. 2018 Aug 7;37:186. doi: 10.1186/s13046-018-0847-7 (PMC6081844; doi:10.1186/s13046-018-0847-7)
Supplement: Supplementary file 2 — Figure S1. The full-length sequence of HEGBC. Representative image of PCR products from 5’-RACE and 3’-RACE assays are shown. Figure S2. RIP assay in NOZ cells was performed using RPLP0 specific antibody or negative control IgG. The bound RNA was detected using qRT-PCR with specific primers against HEGBC. Results are shown as mean ± s.d. of 3 independent experiments. ns, not significant by Student’s t test. (DOCX 15 kb) [file 13046_2018_847_MOESM2_ESM.docx]

**Supplementary Figure Legends**

**Additional file 2: Figure S1** The full-length sequence of HEGBC. Representative image of PCR products from 5'-RACE and 3'-RACE assays are shown.

**Additional file 3: Figure S2** RIP assay in NOZ cells was performed using RPLP0 specific antibody or negative control IgG. The bound RNA was detected using qRT-PCR with specific primers against HEGBC. Results are shown as mean ± s.d. of 3 independent experiments. ns, not significant by Student’s t test.
